# Population Structure and Genotype–Phenotype Associations in a Collection of Oat Landraces and Historic Cultivars

**DOI:** 10.3389/fpls.2016.01077

**Published:** 2016-07-29

**Authors:** Louisa R. Winkler, J. Michael Bonman, Shiaoman Chao, B. Admassu Yimer, Harold Bockelman, Kathy Esvelt Klos

**Affiliations:** ^1^Sustainable Seed Systems Laboratory, Department of Crop and Soil Sciences, Washington State University, PullmanWA, USA; ^2^Small Grains and Potato Germplasm Research Unit, United States Department of Agriculture – Agricultural Research Service, AberdeenID, USA; ^3^Cereal Crops Research Unit, United States Department of Agriculture – Agricultural Research Service, FargoND, USA

**Keywords:** historic oat germplasm collection, association mapping, population structure, genetic diversity, lemma color, oat breeding history

## Abstract

Population structure and genetic architecture of phenotypic traits in oat (*Avena sativa* L.) remain relatively under-researched compared to other small grain species. This study explores the historic context of current elite germplasm, including phenotypic and genetic characterization, with a particular focus on identifying under-utilized areas. A diverse panel of cultivated oat accessions was assembled from the USDA National Small Grains Collection to represent a gene pool relatively unaffected by twentieth century breeding activity and unlikely to have been included in recent molecular studies. The panel was genotyped using an oat iSelect 6K beadchip SNP array. The final dataset included 759 unique individuals and 2,715 polymorphic markers. Some population structure was apparent, with the first three principal components accounting for 38.8% of variation and 73% of individuals belonging to one of three clusters. One cluster with high genetic distinctness appears to have been largely overlooked in twentieth century breeding. Classification and phenotype data provided by the Germplasm Resources Information Network were evaluated for their relationship to population structure. Of the structuring variables evaluated, improvement status (cultivar or landrace) was relatively unimportant, indicating that landraces and cultivars included in the panel were all sampled from a similar underlying population. Instead, lemma color and region of origin showed the strongest explanatory power. An exploratory association mapping study of the panel using a subset of 2,588 mapped markers generated novel indications of genomic regions associated with awn frequency, kernels per spikelet, lemma color, and panicle type. Further results supported previous findings of loci associated with barley yellow dwarf virus tolerance, crown rust (caused by *Puccinia coronata* f. sp. *avenae*) resistance, days to anthesis, and growth habit (winter/spring). In addition, two novel loci were identified for crown rust resistance.

## Introduction

Oat (*Avena sativa* L., 2*n* = 6x = 42) has played an important role in the development of agriculture around the world, but received less investment from the plant and agricultural research sectors during the twentieth century than other small grain crops such as wheat and barley (Frey, 1996). The oat crop has consequently shown slower improvement than wheat and barley in its yield potential and other agronomic traits, and its genetics are less well understood. In recent years, valuable studies addressing the shortfall in oat genetics research have been published. These include studies applying molecular techniques to the characterization of oat genetic diversity, population structure and genotype-phenotype associations (e.g., Kianian et al., 1999; Holland et al., 2002; Baohong et al., 2003; Wight et al., 2004; Newell et al., 2011, 2012; Tanhuanpää et al., 2012; Tinker et al., 2014; Esvelt Klos et al., 2016).

Evidence from existing studies of mid- to late-20th century samples of oat genotypes suggest that cultivated oat populations show high diversity. In a study of 83 North American oat cultivars using restriction fragment length polymorphisms (RFLPs), polymorphism was found to be 54.8% in oat as compared to 5% in wheat and 28% in barley (O’Donoughue et al., 1994), supporting an earlier finding based on pedigree analysis that diversity in North American oat germplasm was greater than that in wheat (Souza and Sorrells, 1989). This diversity is a valuable resource for agriculture because it enables oats to be adapted to a wide range of environments and end-uses.

Population structure in oat has been found to be weak, without any clear morphological source. In populations of barley (*Hordeum vulgare* L.), genotypes fall into distinct sub-populations based on row number (2 vs. 6), growth habit (winter vs. spring) and geographical origin (Hamblin et al., 2010; Pasam et al., 2012), and to some extent also end use (malting vs. feed) (Zhou et al., 2012). In oat, morphological traits such as hull type (hulled or hulless), lemma color and panicle type have not been found to contribute significantly to genetic clustering patterns (Achleitner et al., 2008; Newell et al., 2011).

Instead, the practical differentiation of ‘fall sown’ and ‘spring sown’ oat gene pools in 20th century breeding has been identified as a source of structure in analyses of a global oat panel by Esvelt Klos et al. (2016) and of North American panels by Souza and Sorrells (1991a,b) and Phillips and Murphy (1993). This differentiation was adopted during the 20th century to describe the way in which germplasm is managed by breeding programs: ‘fall sown’ genotypes are those developed for overwinter production in subtropical regions with temperate climates and are generally bred as forage or cover crops, while ‘spring sown’ genotypes are adapted for spring planting and are the focus of most breeding programs at more northerly latitudes. Phillips and Murphy (1993) suggested that the ‘fall sown’/‘spring sown’ distinction became more important over time due to the segregation of genotypes into separate breeding pools. However, the precise genetic basis for the distinction is unknown; and in the literature, there is overlap between it and (i) the ‘red’ vs. ‘common’ oat distinction; (ii) the ‘winter’ vs. ‘spring’ oat distinction; and (iii) the distinction of *Avena sativa* vs. *A. sativa* ssp. *byzantina* C. Koch.

Comparison of material capturing a large range of oat genetic diversity and predating the influence of 20th century breeding with more recent material may be able to reveal whether the ‘fall sown’/‘spring sown’ distinction is informative in the wider oat population and if not, what other characteristics defined population structure before the development of elite germplasm pools during the 20th century. Enhanced understanding of the relationship of modern breeding material to ancestral germplasm is needed if breeders are to more effectively exploit diversity in cultivated oat. Progress toward this goal is becoming increasingly important, given indications of loss of oat diversity from several geographical regions (Baohong et al., 2003; Fu et al., 2003; Achleitner et al., 2008).

Underinvestment in the genetic analysis of oat relative to that of other crops means that large gaps remain in our knowledge of the oat genome. Many aspects of panicle and floret morphology have yet to be genetically characterized, and there is an on-going need for the discovery of alleles conferring disease resistance from under- or unutilized gene pools.

The purpose of the present study was to evaluate population structure and explore genotype-phenotype associations across a broad range of traits in oat germplasm that has been relatively unaffected by 20th century breeding activities. The panel consisted of 759 cultivated oat accessions from the USDA National Small Grains Collection (NSGC) representing cultivars collected before 1930, and landraces collected through the 19th and 20th centuries. Genotyping was performed using a SNP array developed for hexaploid oat by Tinker et al. (2014) and phenotype data was obtained from the USDA’s Genetic Resources Information Network (GRIN)^1^.

## Materials and Methods

### Plant Materials

One thousand temporally and geographically diverse *A. sativa* L. accessions were selected from among the 9,283 available from the NSGC in 2013. The sample was composed of (1) 383 accessions selected from either cultivars or from accessions of uncertain improvement status that had entered the NSGC before 1930; and (2) 617 accessions selected from among all available landraces. Cultivars that entered the NSGC before 1930 are likely to be representative of the same population structure as the landraces, because improved varieties of that era were largely selections from landraces or selections from crosses combining two to four landraces (Stanton, 1936). Cultivars and accessions of uncertain improvement status that entered the NSGC later than 1929, and accessions classified as breeding lines, were excluded to minimize potential cryptic relatedness among lines.

### Genotypes

A single plant from each accession was grown in the greenhouse at Aberdeen, ID, USA in 2013. DNA for genotyping was extracted from young leaves using the cetyl trimethyl ammonium bromide (CTAB) protocol (Anderson et al., 1992) with modifications including use of a bead grinder FastPrep homogenizer (MP Biomedical) for 5 min at 25 strokes/s to homogenize tissue.

Genotypes for 4,975 SNPs were acquired using an oat iSelect 6K beadchip array (Illumina, San Diego, CA, USA) at the USDA-ARS Genotyping Laboratory at Fargo, ND, USA. SNP genotype calls were made with GenomeStudio v2011.1 (Illumina, San Diego, CA, USA) with GenCall set at 0.15. In addition, SNP calls were manually inspected and adjusted in GenomeStudio. Multi-allelic and monomorphic SNPs were eliminated, as well as those with poor genotype calls due to weak signal or ambiguous clustering. There were 2,715 SNPs with no more than 5% missing calls and/or heterozygosity, and at least 5% minor allele frequency (MAF). Genotypic data is deposited in the public database T3/oat^2^.

### Screening for Duplicates

Single nucleotide polymorphism data were additionally used to screen for duplicates within the panel. Three hundred and twenty-four lines were identified as duplicates with one or more other lines at a threshold of 99.5% identity. A single line was retained from each set of duplicates based on completeness of genotype data. An additional accession was removed due to unidentified problems in the genotype coding. The final sample had 759 accessions.

### Phenotypes

Phenotypes were downloaded from the GRIN^3^ on September 22, 2014 and are representative of the entire NSGC accession rather than the single plants used for genotyping. For that reason, phenotypes recorded in GRIN as a range or as mixed were considered missing data. In all but rare cases, GRIN data are based on unreplicated experiments. Country of origin was used to assign each accession to a region of origin as defined by the United Nations Statistics Division^4^.

Detailed descriptions of phenotyping methods are available at http://www.ars-grin.gov/npgs/searchgrin.html. In brief: Awn frequency, hullessness of the threshed grain, amount of plant lodging near maturity, panicle density, panicle type, shattering, and straw breakage at maturity were assessed by visual observation. Beta glucan, lipid, and protein content of the kernel were expressed on a percent dry weight basis. Reaction to BYD virus was evaluated on a 1-9 scale with 1 = resistant and 9 = susceptible. Crown rust (*Puccinia coronata*) reaction was to a naturally occurring mixture of races in the Saint Paul, MN buckthorn nursery. Reactions rated highly resistant, resistant, moderately resistant to resistant, moderately resistant, moderately resistant to moderately susceptible, moderately susceptible, moderately susceptible to susceptible, and susceptible were converted to a numerical score from 0 to 7 for analysis. Crown rust severity was assessed as the percentage of leaf area affected per plot. Reaction to greenbug (*Schizaphis graminus*) was assessed as the percent of the resistant check ‘Andrew’. Growth habit was assessed after spring planting in Aberdeen, ID, USA as facultative, spring, winter, or a mix of types. However, we used only those deemed spring or winter for analysis. Days to anthesis was recorded as the Julian calendar date at which 50% of spikes are fully emerged, following spring sowing. Kernels per spikelet and spikelets per panicle were averaged over five panicles. Lemma color was evaluated using a Foss GrainCheck^TM^ 2312 Analyzer (Foss Tecatur AB, Hillerød, Denmark). Lemma color was coded based on GC color intensity where 1 < 46.00 (black), 4 = 46.01 to 57.99 (gray), 6 = 58.00 to 71.99 (red), 9 = 72.00 to 82.99 (yellow), and 8 > 83.00 (amber/white). Panicle length was taken as the average distance (in cm) between the lowest node and the tip of the apical spike over five panicles. Plant height was taken as the average distance (in cm) from the ground to the panicle tops. Reaction to smut was recorded as the amount of the combination of covered smut (*Ustilago kolleri*) and loose smut (*U. avanae*) expressed as a percent of the susceptible check. Straw color was recorded as 1 = black, 2 = blue, 3 = brown, 5 = purple, 7 = tan, 8 = white/amber, and 9 = yellow. Test weight of grain was reported in pounds per bushel. Yield was recorded as the weight of harvested grain in grams per unreplicated 2.44 m row.

### Population Structure and Cluster Analyses

Principal component analysis (PCA) was performed in TASSEL v.4.0 (Bradbury et al., 2007). Principal coordinate analysis (PCoA) to visualize patterns of diversity was performed using the GenAlex software package (Peakall and Smouse, 2006, 2012). Analysis of molecular variance (AMOVA) to partition genetic diversity within and among populations classified according to a range of available variables was performed using Powermarker software version 3.25 (Liu and Muse, 2005).

STRUCTURE software version 2.3.4 (Pritchard et al., 2000) was used for model-based (Bayesian) exploration of population structure. The full set of 2,715 markers was included since there was no detectable pattern in the distribution of missing data. The admixture model was chosen. Initially, twenty independent simulations per *K* value (number of population clusters) were run for *K* = 1 to 12. Burn-in was set to 10,000 and Monte-Carlo Markov Chain replications (MCMC) to 25,000 after burn-in. Values of *L(K)* (log posterior probability of the data) returned by STRUCTURE were averaged across simulations for each *K* value. Following the method of Evanno et al. (2005), a plot of the second order rate of change in *L(K)* between values of *K* (Δ*K*) was created. A further run with burn-in = 100,000 and 100,000 MCMC replications was performed to assign individuals to population clusters using the value of *K* inferred from the initial run. A threshold of 80% genetic membership coefficient was used to assign genotypes to a specific cluster, below which they were designated ‘admixed’.

Using the relationship matrix function in JMP Genomics, distance-based hierarchical cluster analysis was done by first generating a similarity matrix based on allele sharing for each accession pair using the subset of SNPs without missing genotype calls. Accessions were then grouped by the Fast Ward hierarchical clustering procedure.

Distribution of phenotypic or origin categories across inferred population clusters was explored by calculating expected distribution based on random allocation of category members to clusters, and calculating over- or underrepresentation based on deviation from expected values as a proportion of group membership. Observations were generally noted if they exceeded 15% deviation from expected values. Statistical significance of results was tested by the chi-squared test. For evaluation of patterns in year of collection, accessions were grouped by decade of collection to avoid excessively small cell sizes in the analysis. Because classification as ‘fall sown’ vs. ‘spring sown’ is not undertaken in GRIN, classification as ‘red’ vs. ‘common’ type and winter vs. spring growth habit were evaluated as possible substitutes.

### Genome Wide Association

For the majority of GRIN phenotypes, observations for each accession were taken in a single study environment with no accessions common to multiple study/environments which could be used to estimate environmental effects. Because of this, association analyses were performed within study environments and only for those with *N* > 80 accessions in the panel.

Single nucleotide polymorphism locations were taken as those on the oat consensus map version 3.1 reported by Chaffin et al. (2016). In brief, these authors developed dense linkage maps for 12 bi-parental populations, all of which had been genotyped using the Oat 6K custom Illumina Infinium iSelect BeadChip assay. In addition, six of the populations had also been genotyped using two Illumina SNP Oligo Pooled Assays, one had been genotyped using the wheat 90K, and seven had been genotyped using genotype-by-sequencing technology. Previously mapped markers of various other technologies (RFLP, SSR, AFLP) were included in the Kanota × Ogle and Ogle × TAM O-301 linkage maps. An iterative approach was used to identify homeologous linkage groups across the 12 genetic maps. Clusters of conserved collinearity identified 21 chromosomes, assigned labels “Mrg01” through “Mrg33”, with “Mrg” standing for “merged LG”. Within linkage groups, a smoothing algorithm was used to assign average map distances between markers. In this way, the position of SNPs investigated in the present study could be compared with those of previously investigated markers. Of the 2,715 genotyped markers used in the present study, 2,588 are mapped on the consensus map and could therefore be included in the GWAS.

Genotype-phenotype association was evaluated using a mixed linear model (MLM) procedure in TASSEL v4 (Bradbury et al., 2007) under the default settings. Population structure was accounted for by incorporating the first three PC as covariates in the model. Cryptic relatedness among lines was accounted for using a kinship matrix. The ability of the MLM to adjust the tendency for inflation of the type I error rate was assessed by examining the quantile-quantile (Q-Q) plots. Only markers with map positions (2,588) were included in the analysis. This number of markers was used to establish the threshold for declaring statistical significance of associations at *p* ≤ 1.93 × 10^-5^, calculated by applying the Bonferroni correction method with an experiment-wise alpha level set at 0.05.

## Results

### Population Structure and Cluster Membership

Compared with the NSGC *A. sativa* collection, the panel compiled for this study had proportionally fewer cultivars and accessions of uncertain improvement status compared with landraces. This distribution is undoubtedly due to the fact that we limited our selection of cultivars to those entered before 1930. Within the landraces, the present sample maintains the collection year and region of origin distributions of the NSGC (Supplementary Figures S1A,B).

**FIGURE 1:**
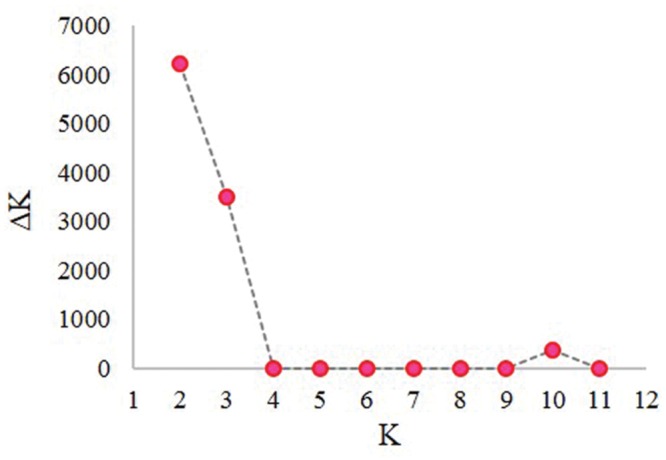
**Plot of change in likelihood of the data, *L(K)*, at values of *K* from 1 to 11, used to infer the true value of *K* following the method of Evanno et al. (2005)**.

**FIGURE 2:**
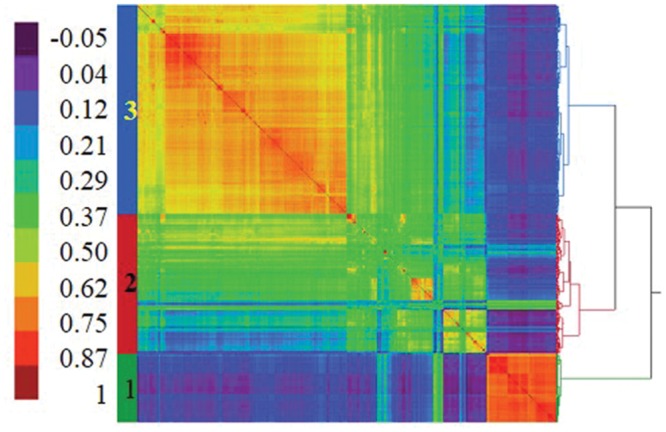
**Population structure of a 759-member panel of historic and unimproved accessions of cultivated oat visualized by hierarchical clustering with Ward’s method using a genetic similarity matrix generated from SNP data.** ^a^The heat plot was constructed based on allele -sharing coefficients calculated in the relationship matrix function in JMP Genomics v. 7.1. The heat plot shows pairwise marker-to-marker allele sharing between two accessions with red representing the highest proportion of shared alleles and blue indicating the lowest. Three clusters (subpopulations) are shown by different colors bars to the left and numbered by their equivalent clusters as assigned by STRUCTURE software.

The first three principal components (PC) accounted for 23.6, 11.8, and 3.4% of marker variation, respectively (a total of 38.8%). The first five PC together accounted for 43.3% of genetic variation among accessions. A plot of the eigenvalues of the first 25 PC suggested that the first three PC account for most of the population structure in this panel (not shown). Similar results were obtained from the model-based analysis where the plot of *K* vs. Δ*K* (**Figure 1**) showed a rapid reduction in Δ*K*, normally regarded as a signal that the true value of *K* has been reached, between *K* = 3 and 4. A further run of STRUCTURE was used to assign accessions in the panel to three population clusters (**Table 1**). Seventy-three percent of accessions exceeded the 80% membership threshold and could be assigned to a specific cluster, while 27% were considered admixed. Cluster 1 was more divergent from 2 or 3 than they were from each other, based on indicators from both STRUCTURE (allele frequency divergence, not shown) and hierarchical clustering (allele sharing; **Figure 2**). Cluster 2 contained the fewest members but showed the highest within-group gene diversity of all clusters (**Table 1**). Overlap between cluster assignments based on PCA, hierarchical clustering, and model-based analyses confirmed that all detected the same population structure. STRUCTURE-assigned groupings were used for further evaluation of origin and phenotypic trait distribution across population clusters.

**Table 1:** Membership and within-cluster diversity of subpopulations in a 759-member panel of oat accessions, assigned by model-based clustering in STRUCTURE software.

Cluster	1	2	3	Ad-mixed
Number of members	124	77	355	203
% of sample	16	10	47	27
Gene diversity^a^ within cluster	0.0978	0.2505	0.1753	-

*^a^Gene diversity is a measure of within-cluster polymorphism across all markers, calculated as*

*${\text{H}}_{\text{x}}\text{=}\frac{1}{L}\sum_{L=1}^{L}[1-\sum_{j=1}^{Ji}{\hat{p}}_{x,j}^{(i)2}]$*

*Where *L* is the number of loci (markers), *J_*i*_* the number of alleles at locus *i* and ${\hat{p}}_{x,j}^{(i)2}$ is the posterior mean estimated allele frequency of allele *j* at locus *i* in population *x*. Further details are available in STRUCTURE software documentation (Pritchard et al., 2010).*

AMOVA was used to compare a range of variables for their power to explain genetic variability in the panel (**Table 2**). Assignment to the STRUCTURE groupings accounted for the largest proportion of among-group genetic variance. Lemma color and region of origin groups showed some importance as explanatory variables, while growth habit and classification as ‘red’ or ‘common’ oat were far less important. Improvement status groups accounted for only 2.5% of variance, suggesting that that landraces, unimproved material, and cultivars are all drawn from the same underlying population structure.

**Table 2:** Genetic variance partitioned according to different population classification approaches for 759 oat accessions, calculated from AMOVA.

Grouping variable	Among-group variance (%)
Cluster (STRUCTURE)^a^	37.3
Lemma color^b^	13.8
Region of origin^c^	13.1
Growth habit^d^	7.9
Red vs. common^e^	7.4
Improvement status^f^	2.5

*^a^Clusters were assigned using STRUCTURE software; ^b^lemma color is evaluated using the Foss GrainCheck analyzer; ^c^region of origin is based on country of collection, classified by region according to the UN system; ^d^growth habit is classified as ‘spring’ or ‘winter’; ^e^‘red’ vs. ‘common’ oat refers to traditional classification based on fracture type and other characteristics; ^f^improvement status is ‘landrace’, ‘cultivar,’ or ‘uncertain’. See “Materials and Methods” for details.*

Improvement status, region of origin (collection site), growth habit (spring, winter, or facultative), lemma color, and ‘red’ vs. ‘common’ classification were explored for their association with STRUCTURE population clusters. The chi-squared test showed non-random distributions for all traits studied (*p* < 0.01).

Cluster 1 was characterized as coming predominantly from Western Asia (46% of members, 23% above expectation), Southern Europe and Northern Africa (**Table 3**); cluster 2 as widely distributed across Central Asia and Europe; and cluster 3 as predominantly European and North American. North American accessions, which account for 18.8% of panel members, are overrepresented in the admixed group and underrepresented in cluster 1. Cluster 1 was almost entirely composed of landraces (**Table 4**). ‘Spring’ growth habit was predominant across the whole panel (78% of rated entries), but those accessions designated ‘winter’ appeared highly disproportionately in cluster 1, somewhat disproportionately in cluster 2, and were underrepresented in cluster 3 (**Table 4**).

**Table 3:** Distribution of 759 oat landraces and historic cultivars across regions of origin (collection site) categories expressed as the observed % membership of population clusters (O) and the % deviation from expected value (E) based on a random distribution.

	Population cluster^b^								Sum across clusters, % panel
	Admixed		1		2		3		
Region of origin^a^	O	O-E/ E (%)	O	O-E/ E (%)	O	O-E/ E (%)	O	O-E/ E (%)	
Central Asia	1	+7	0	-100	1	+41	1	+22	0.9
Eastern Africa	3	+74	2	-18	6	+229	0	-86	2.0
Eastern Asia	2	-38	0	-100	0	-100	6	+78	3.2
Eastern Europe	4	-60	1	-93	6	-41	19	+76	11.1
North America	32	+67	2	-87	6	-66	20	+6	18.8
Northern Africa	1	-50	9	+349	3	+31	0	-100	2.0
Northern Europe	8	+32	0	-100	10	+64	6	+2	6.3
Oceania	11	+139	2	-49	1	-73	3	-47	4.7
South America	4	-17	7	+53	17	+256	2	-64	4.7
Southern Africa	0	-100	1	+206	0	-100	0	+7	0.3
Southern Asia	3	+74	2	+22	1	-34	1	-43	2.0
Southern Europe	3	-84	27	+27	16	-28	31	+45	21.6
Western Asia	23	+22	46	+142	19	+3	7	-63	19.0
Western Europe	3	-14	0	-100	12	+241	3	-10	3.4

*^a^Region of origin describes where an accession was collected and is according to classification of countries by the UN Statistics Division. ^b^Population clusters assigned according to model-based clustering in STRUCTURE software.*

**Table 4:** Summary of selected phenotypic trait values by population cluster in a panel of 759 oat landraces and historic cultivars.

		Population cluster^c^				Whole-panel mean
		Admixed	1	2	3	
Kernel weight (g)	Mean	30.2	37.5	29.0	29.1	30.8
	SD	5.5	4.6	4.4	3.8	5.4
Growth habit^a^, % of cluster	Spring	90	30	53	92	78
	Winter	10	70	47	8	22
Lemma color^b^, % of cluster	Black	19	3	12	3	6
	Gray	1	3	7	1	1
	Red	26	84	14	9	23
	White	4	0	5	4	3
	Yellow	51	10	63	83	55
Improvement status, % of cluster	Landrace	50	93	60	69	67
	Cultivar	24	2	17	14	15
	Uncertain	26	6	23	17	18
Cluster members as % of panel		27	16	10	47	

*^a^Accessions scored as Spring or Winter based on flowering time after spring sowing. ^b^Lemma color is evaluated using the Foss GrainCheck analyzer. ^c^Clusters are those assigned by STRUCTURE software.*

**FIGURE 3:**
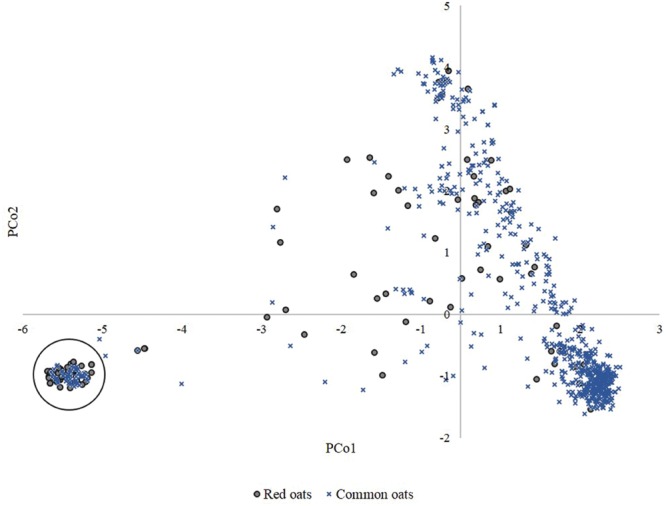
**Principal coordinates 1 vs. 2 plotted for 759 oat accessions and colored according to classification as ‘red’ or ‘common’ oat.** Cluster 1 is circled.

Evaluation of lemma color showed that yellow-hulled oats were disproportionately likely to come from cluster 3, which was also less likely to be a source of black, gray, or red lemma colors (**Table 4**). Black oats were in excess in the admixed group, while gray lemma color was in excess in clusters 1 and 2 and red color in cluster 1. There was imperfect correspondence between accessions with red lemma color and those classified as ‘red’ type oats: 61% of accessions with red lemma color were classified as ‘common’ oat, and 38% of all oats classified as ‘red’ type had yellow, gray or black lemma color. A PCo plot colored by the ‘red’/‘common’ classification (**Figure 3**) suggested that ‘red’ types cover a similar extent of genetic space to that covered by ‘common’ accessions, but are concentrated in cluster 1 (38% overrepresentation at *p* < 0.01). Cluster 1 consists of 59% ‘red’ oats, and 54% of all accessions classified as ‘red’ type belong to this cluster.

Given the importance of both lemma color and region of origin as structuring variables, co-segregation between them was explored. **Table 5** shows that an excess of black-colored oats was collected in Northern and Western Europe, while red-colored oats came primarily from Northern Africa and Southern Asia. Accessions from Western Asia show relatively well-balanced representation of lemma colors. Results from gray and white color categories should be interpreted with caution since the number of accessions in either category was very small.

**Table 5:** Distribution of 677 panel members classified for lemma color across their regions of origin; values represent deviation of observed as percentage of expected number per cell.

Region of origin^a^	Lemma color^b^					Total accessions by region
	Black	Gray	Red	White	Yellow	
		
	% over- or underrepresentation					
Northern Africa	+18	-100	+187	-100	-73	12
Eastern Africa	+101	-100	+64	-100	-30	7
Southern Africa	-100	-100	+91	-100	-19	2
North America	-9	-50	-57	+228	+13	124
South America	+76	+669	+20	-15	-34	32
Central Asia	+101	-100	-100	-100	+39	7
Eastern Asia	+92	-100	-13	-100	+4	22
Southern Asia	-100	-100	+109	-100	-26	11
Western Asia	-17	+81	+94	-80	-35	136
Northern Europe	+237	+34	-100	-100	+20	46
Eastern Europe	-45	-100	-55	+146	+23	77
Southern Europe	-100	-100	+4	-100	+18	147
Western Europe	+370	-100	-84	+13	-5	24
Oceania	-100	+105	+27	-100	+3	30

Total accessions by color	48	11	177	25	416	

*^a^Region of origin describes where an accession was collected and is based on classification of countries according to the UN Statistics Division. ^b^Only those accessions for which GRIN records a single lemma color value were included.*

Cluster 1 showed substantially higher mean kernel weight than other clusters (**Table 4**).

### Genome-Wide Association

Sample size and phenotypic distributions (mean and standard deviation) of traits used in association analyses are presented in Supplementary Tables S1.1 and S1.2. It must be emphasized that different studies of the same trait generally contain non-overlapping groups of individuals which may therefore differ in their alleles and patterns of LD. Statistically significant associations were observed for crown rust reaction and severity, BYDV reaction, awn frequency, kernels per spikelet, lemma color, panicle type, days to anthesis, and growth habit (**Table 6**). Supplementary Figures S2A-I present a visual summary of these findings, while the text below focuses on those traits of most relevance to the present study.

**Table 6:** Single nucleotide polymorphisms associated with GRIN phenotypes at *p* ≤ 1.93 × 10^-5^ and their positions in the oat consensus map (see Supplementary Table S1.1 for the number of accessions included in each study/environment).

Phenotype	Study/environment	SNP	Linkage Group	Position (cM)	*P*-value
Awn frequency	Aberdeen 84	GMI_GBS_97872	Mrg17	31.1	1.60E-6
	Aberdeen 85	GMI_GBS_94371	Mrg02	28.1	1.72E-5
BYDV	Urbana 84	GMI_ES17_lrc19617_111	Mrg17	70.2	1.93E-6
		GMI_GBS_71132	Mrg17	70.2	5.84E-6
		GMI_DS_LB_10400	Mrg17	70.5	7.32E-8
Crown rust reaction	Saint Paul 94	GMI_ES_CC10897_223	Mrg08	131.3	3.57E-6
		GMI_DS_LB_6161	Mrg23	53.8	6.33E-6
		GMI_ES05_c16268_365	Mrg28	35.9	2.91E-6
		GMI_ES17_c6857_627	Mrg28	44.4	6.33E-6
	Saint Paul 97	GMI_ES14_c576_821	Mrg01	74.7	3.40E-6
		GMI_GBS_92025	Mrg20	135.8	1.36E-5
Crown rust severity	Saint Paul 97	GMI_GBS_1448	Mrg08	82.4	1.34E-5
Days to anthesis	Aberdeen 83	GMI_ES03_c7453_413	Mrg02	60.4	1.20E-5
Growth habit	Aberdeen 96	GMI_DS_LB_4706	Mrg06	65.3	2.84E-6
Kernels per spikelet	Aberdeen 85	GMI_GBS_69423	Mrg20	181.2	8.82E-6
Lemma color	Aberdeen 11	GMI_ES15_c2369_181	Mrg20	14.7	2.45E-27
Panicle type	Aberdeen 83	GMI_ES05_lrc12954_281	Mrg01	11.3	8.15E-8
		GMI_ES02_c13759_273	Mrg01	12.9	2.29E-7
		GMI_GBS_62243	Mrg09	104.7	1.16E-5
	Aberdeen 84	GMI_ES14_c967_122	Mrg15	33.1	1.19E-5
		GMI_ES22_c10247_408	Mrg20	202.6	1.27E-6
	Aberdeen 85	GMI_ES02_c7295_356	Mrg11	50.5	5.71 E-7

A cluster of SNPs at 70.2 to 70.5 cM on Mrg17 was associated with reaction to BYDV evaluated in Urbana 84, but not in Urbana 85 (**Table 6**; Supplementary Figure S2B). Of the SNPs in this region, GMI_GBS_71132 at 70.2 cM, significant in Urbana 84, had the lowest *p*-value in Urbana 85 (*p* = 0.036), providing a true replicate for this effect.

As expected given the highly variable nature of the mixture of crown rust races generated in the Minnesota buckthorn nursery at St Paul, association with reaction to crown rust and crown rust severity were not consistent across study/environments (**Table 6**; Supplementary Figures S2C,D). Evidence of association within a single environment was detected on five linkage groups.

A QTN for days to anthesis (heading date/flowering time) was detected on Mrg02 at 60.4 cM in Aberdeen 83 (Supplementary Figure S2E). Weaker evidence (*p* < 0.01) of this marker-trait association was observed in Aberdeenr 84 (not shown). The same marker was also associated at *p* < 1.0 × 10^-4^ with crown rust reaction and severity in Saint Paul 97, and with awn frequency in Aberdeen 83.

Variation in growth habit was significantly associated with a marker mapping to 65.3 cM on Mrg06. Spring classification describes 90% of lines carrying the major allele and 27% of lines with the minor allele. Weaker evidence of association with growth habit was present at several additional regions (Supplementary Figure S2F).

A single SNP on Mrg20 was strongly associated with lemma color. At that locus, genotypes carrying the major allele were predominantly yellow (82.4%), whereas those carrying the minor allele were more likely to be either red (31.6%) or black (21.1%).

## Discussion

### New Perspectives on Population Structure in Cultivated Oat

Studies of the global gene pool of cultivated oat have generally reported weak structure. PC1-5 accounted for 23.8% of genetic variation in the 635-member CORE panel of elite cultivars (Esvelt Klos et al., 2016), and 22.3% in a 1,205-member global panel (Newell et al., 2011). A study of the core subset of the NSGC barley accessions, considered to be more strongly structured, found that the first four PCs explained 26.4% of variation (Muñoz-Amatriaín et al., 2014). In the present study, PC1-3 alone explained 38.8% of marker variation, and the majority of the population appears to cluster into three subgroups.

The lack of correspondence between these subgroups and classification as ‘landrace’ or ‘cultivar’ (explaining only 2.5% of genetic variation in AMOVA) reflects the early historical era during which cultivars in the panel were collected. Cultivars in the present study all entered the NSGC prior to 1930. In the late nineteenth and early twentieth centuries, oat cultivars tended to be selections from landraces rather than artificial crosses, and breeding programs with highly managed germplasm pools did not yet exist.

Lemma color was strongly associated with population structure as quantified by AMOVA (13.8%). Indications from earlier quantitative work (e.g., Coffman, 1964, see below) and from GWAS in the present study are that lemma color is under relatively simple genetic control by a small number of loci. A similar situation is observed in barley, where row-type (2-row vs. 6-row) is a single-gene trait strongly associated with population structure (Muñoz-Amatriaín et al., 2014). One possible explanation is that different lemma colors arose in different populations/genetic backgrounds are therefore associated with geographical differentiation. Prevalence of black-colored oats from the cooler climates of northern and western Europe (the majority of these genotypes were collected in the UK and France) and of red-colored oats from the warmer climates of Africa and Southern Asia suggests a possible relationship between climate-adaptation traits and lemma color. Distribution of lemma color by region also reflects cultural preference and selection. Lemma color categories show relatively balanced representation amongst accessions from Western Asia (**Table 5**), which is both an important center of diversification for *Avena* sp and a region where they are less important as a cultivated crop (Leggett and Thomas, 1995). Accessions from this region may reflect greater influence of natural diversity over human preference than in other regions where oat has been subjected to more active selection. If so, it attests to the strong selection against red, gray, and black hull colors in most oat breeding areas which has resulted in the complete absence of these colors from the 635 members of the global CORE elite panel, a product of 20th century breeding (unpublished work by authors).

Region of origin (collection site) accounted for 13.1% of genetic variation by AMOVA, a similar proportion to that captured by lemma color. Its importance to population structure in the present study echoes the findings of Fu et al. (2005) in oat and Bonman et al. (2015) for a panel of 3,230 worldwide bread wheat accessions. Gene bank records of region of origin for panel members in the present study may indicate the region in which a genotype evolved or one to which it was later transported by humans; if the latter, it may indicate that a genotype is preferred for cultivation or is maintained as a potential germplasm resource rather than for its strong regional adaptation. Were it possible to more finely resolve the classification of origin or cultural preference, these categories could prove to hold greater explanatory power than that shown by region of origin as evaluated here.

Esvelt Klos et al. (2016) found that 16% of variation in the CORE panel of 635 elite accessions was accounted for by ‘spring sown’/‘fall sown’ classification, a distinction not present in GRIN. Examination of GRIN data, in addition to variety descriptions offered by Stanton (1955) and Coffman (1977), show that while there is a tendency for red oats in practice to be fall sown and therefore sometimes to be known as ‘winter oats’, there is also no established relationship of cold tolerance and/or vernalization response with one or the other classification. The finding that growth habit (spring/winter) and oat type (‘red’/‘common’) appear to be relatively uninformative of population structure in the present study suggests twentieth century breeding activity has influenced the ‘spring sown’/‘fall sown’ distinction found to account for population structure in modern cultivated oat germplasm, as previously indicated by Phillips and Murphy (1993). It cannot, however, be regarded as conclusive evidence, since (i) the classifications may not be fully correspondent, and/or (ii) the classification of growth habit in GRIN may not be accurate (discussed below).

The population structure of the germplasm panel used in our study clearly shows a genetic cluster that has largely been overlooked in 20th century breeding. Cluster 1 was characterized by high genetic distinctness and relatively low internal diversity, with a high proportion of red and winter oats. A similar cluster was identified in a previous survey of global oat germplasm by Newell et al. (2011). Of the important progenitor varieties in US oat breeding identified by Coffman (1977, p. 63), many are included in the present study; none, however, originates from cluster 1. A comparison of the 759 accessions in the present study with modern elite germplasm of the oat CORE collection using the same SNP chip has shown that cluster 1 is genetically distinct from modern germplasm (unpublished work by authors). This finding helps to explain the net reduction of allelic diversity in cultivated oat during the 20th century in Canada demonstrated by Fu et al. (2003). Cluster 1 could yield valuable new disease resistance or agronomic trait alleles, and more detailed evaluation of this material is warranted. Higher average kernel weight of cluster 1 (37.4 g) in comparison with clusters 2 and 3 (29.0 and 29.1 g, respectively) makes it a particularly promising source of grain–oat germplasm.

### Genome-Wide Association Study

### Linkage Disequilibrium and Power of the Study Design

The power and resolution of GWAS is influenced by LD between markers in the population studied, and every population is associated with its own unique pattern of LD. In a worldwide panel of 635 elite breeding lines, Esvelt Klos et al. (2016) estimated that LD of *r*^2^> 0.10 was observed within linkage groups at an average distance between markers of 0.44 cM. The same value was estimated as 2.5 cM by Newell et al. (2011) for 1,405 breeding lines and landraces. In both of these studies, naïve estimates gave considerably inflated LD values, underlining the importance of accounting for population structure and kinship in genetic analysis of oat. However, provided that such measures are taken, Newell et al. (2011) showed that intermixing between population clusters found to occur in oat is sufficient to justify conducting GWAS with germplasm of diverse origins and backgrounds.

The same authors suggested in their study that a minimum of one marker every cM be used for GWAS in oat. Chaffin et al. (2016) estimate the length of the oat consensus map at 2,843 cM, such that the marker set of 2,588 available for GWAS in the present study provided an average distance between markers of 1.09 cM. While this level of coverage can be considered appropriate for association mapping, it is unlikely to deliver a high level of power, and this should be taken into account in the interpretation of results.

The use of historical phenotypic data in these analyses resulted in what can be considered several small studies of genotype-phenotype association, each with an independent sample evaluated in a single environment (i.e., two studies of the same trait may involve two different groups of individuals) and without replications. Small sample size, lack of replication within or across environments and the relatively small number of markers available represent limitations on the power of the present study to reliably detect small to moderate SNP effects on phenotype, so we suggest that results should be interpreted as exploratory, with the primary purpose of highlighting genomic regions of interest for future research. For these exploratory GWAS, we chose to apply the same MLM approach to all genotype-phenotype analyses. It is likely that a more nuanced approach in future efforts would improve statistical power to detect QTL for some phenotypes, especially in the case of a skewed distribution or a genetic architecture characterized by rare variants and moderate effect sizes (Yang et al., 2014). Given the described limitations, our discovery of significant associations is encouraging for future efforts.

**FIGURE 4:**
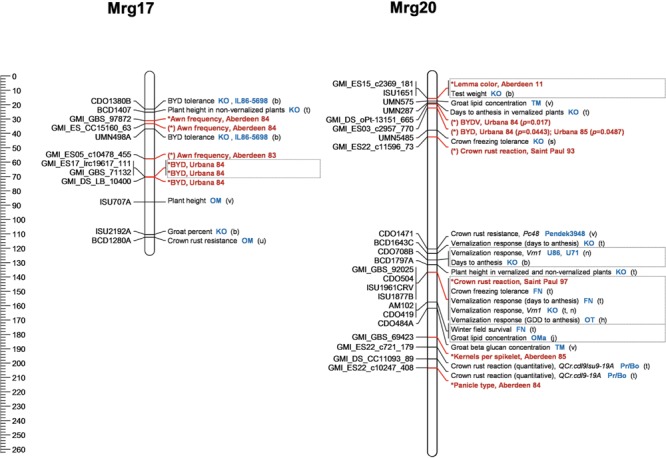
**Two oat linkage groups showing marker-trait associations identified in the present study alongside those identified in previous work.** ^a^Results from the present study are in bold red font, and the environment(s) in which an association was found is recorded. Those marked with ^∗^ were significant at a threshold of *p* < 1.93 × 10^-5^, and those marked with (^∗^) were near-significant at a threshold of 4.0 × 10^-5^ unless otherwise stated. Where previous findings are reported, a designation of the population in which the study was conducted is displayed in blue font, and the reference is given by a lower-case letter in brackets (see overleaf for key to references and population abbreviations). Putative genes are in italics. Where a trait association was mapped to an interval rather than a single marker, the figure displays the marker which showed the strongest association and/or was mapped the closest. Boxes are drawn around associations mapping to the same locus. Stem rust is caused by *Puccinia graminis* f. sp. *avenae*. crown rust by *Puccinia coronata* f. sp. *avenae*. GDD stands for growing degree days. See Supplementary Figures S3A-G for remaining linkage groups. The figure was constructed with the help of MapChart v2.3 (Voorrips, 2002). (See **Table 7** for Refereces).

Association mapping analyses conducted for the present study address several traits which have not been part of recent molecular work in oat and thus represent an initial attempt to discover their underlying genetics. The inclusion of a broad range of traits presents opportunities to look at relationships between them. The recently completed oat consensus map also permits the examination of our findings together with previously identified marker-trait associations. A visualization of the annotated oat genome in **Figure 4** (Mrg17 and 20) and Supplementary Figures S3A-G displays present findings alongside several years’ worth of findings from genetic mapping efforts in oat.

**Table 7:** References for **Figure 4** and Supplementary Figure S3.

Reference	Population used
(a) Babiker et al., 2014	Pr/Bo (ProvenaxCDC-Boyer); Pr/94 (Provenax94197A1-9-2-2-2-5); Bo/94 (CDC-Boyer x 94197A1-9-2-2-2-5)
(b) Barbosa-Neto et al., 2000	KO (Kanota × Ogle)
(c) De Koeyer et al., 2004	TM (Terra × Marion)
(d) Gnanesh et al., 2013	Various genetic backgrounds
(e) Gnanesh et al., 2015	OTM (OT3019 × Morton)
(f) Groh et al., 2001	KO, KM (Kanota × Marion)
(g) Holland et al., 1997	KO
(h) Holland et al., 2002	OT (Ogle × TAMO-301)
(i) Jin et al., 1998	C64/IL86 (Clintland64 × IL86-5698)
(j) Kianian et al., 1999	KO, OMa (Ogle × Marion)
(k) Kianian et al., 2000	KO, OMa (Ogle × Marion)
(l) Lin et al., 2014	AsbMN (AC Assiniboia × MN841801); MedMN (AC Medallion × MN841801); MakMN (Makuru × MN841801)
(m) Locatelli et al., 2006	U8/Pc68 (UFRGS8 × Pc68/5^∗^Starter); U71/Pc68 (UFRGS 88 1971 × Pc68/5^∗^Starter)
(n) Nava et al., 2012	U86 (UFRGS 8 × UFRGS 930605); U71P (UFRGS 881971 × Pc68/5^∗^Starter)
(o) O’Donoughue et al., 1996	OT/Du (OT328 × Dumont); R0/R0Pg9 (Rodney 0 × Rodney 0-Pg9); R0/R0Pg13 (Rodney 0 × Rodney 0-Pg13)
(p) Siripoonwiwat et al., 1996	KO
(q) Wight et al., 1994	Various genetic backgrounds
(r) Wight et al., 2004	Pendek3948 (Pendek-39 × Pendek-48); Pendek4838 (Pendek-48 × Pendek-38)
(s) Wooten et al., 2008	KO
(t) Wooten et al., 2009	FN (Fulghum × Norline)
(u) Zhu and Kaeppler, 2003	OM (Ogle × MAM17-5)
(v) Zhu et al., 2003	OM
(w) Zhu et al., 2004	OM

### BYD

Three markers were linked with BYD tolerance under one of the two environments tested in the present study. They map close together on Mrg17, suggesting a single QTL in this region (**Figure 4**). The region appears to be homologous with one carrying markers for a major BYD tolerance locus identified in separate oat populations by Jin et al. (1998) and Barbosa-Neto et al. (2000). *Ryd3* is a major gene for BYD tolerance in barley which has been mapped to a genetic interval corresponding to a segment of rice chromosome Os02 (Lüpken et al., 2013). Oat Mrg17 shares a large region of synteny with Os02 (Chaffin et al., 2016), raising the possibility that the BYD tolerance QTN identified in the present study may be a *Ryd3* orthologue.

The studies by Jin et al. (1998) and Barbosa-Neto et al. (2000) respectively identified a further 20 and 5 chromosomal regions with linkage to BYD tolerance, and additional loci were identified by Zhu et al. (2003). Inability of the present study to map more than one locus and detection under only one environment may be related to the natural BYD infection used in phenotyping. BYD isolate frequencies are known to vary across time and location (Comeau, 1984; McKenzie et al., 1985), and as with crown rust races, may differ in their virulence genes (Jin et al., 1998). The QTN reported here may be isolate specific, confirmation of which would require further research under controlled conditions.

### Crown Rust

The present study identified five SNPs associated with oat seedling resistance on Mrg01, Mrg08, Mrg20, Mrg23 and Mrg28. Candidate genes for SNPs on Mrg08 and Mrg28 are *PcKM* (which may be *Pc45*) and *Pc91*, respectively (Gnanesh et al., 2013, 2015). The marker on Mrg20 (GMI_GBS_92025) may identify *Pc48*, first mapped by Wight et al. (Wight et al., 2004; **Figure 4**). No candidate genes could be found for seedling resistance markers on Mrg23 and Mrg01, though they could exist among the many *Pc* genes yet to be mapped.

For protection of oats against crown rust, oat breeders have tended to turn to race specific seedling resistance conferred by single genes such as those described above; but the crown rust pathogen is highly variable and has been shown to quickly overcome such resistance (Chong and Kolmer, 1993). Recent years have seen increased interest in partial (quantitative) resistance, for which Babiker et al. (2014) have now identified a number of QTL. One of the QTL they identified maps to Mrg08, close to where the present study found an SNP significantly associated with adult plant resistance. Further investigation is needed to clarify whether the same gene is implicated in both findings.

Wight et al. (2004) also established marker linkages for crown rust resistance gene *Pc38* which situate it at approximately 70 cM on Mrg02 (Supplementary Figure S3A). In the same region, we identified several markers near-significant (*p* < 0.0001) for crown rust reaction and severity and one marker significant for days to anthesis (GMI_ES03_c7453_413). A marker for heading date of unvernalized plants identified by Holland et al. (1997) also locates in this region. In a second example of association between the genetics of flowering time and rust resistance, a marker linked to the vernalization response gene *Vrn1* by Nava et al. (2012) is co-located on Mrg20 with a marker linked by Wight et al. (2004) to rust resistance gene *Pc48*. One explanation for such findings is that developmental stage of the plant influences its susceptibility to the crown rust pathogen.

### Lemma Color

Up to three loci have been observed to control lemma color, depending upon the specific cross, with interactions between loci including epistatic dominance (e.g., black over yellow) and complementarity (e.g., intensification of red color) (Coffman, 1964; Hoffman, 1999). Further, each genome within the full hexaploid set may carry multiple loci contributing to lemma color, as opposed to one locus per genome (Hoffman, 1999). In contrast to these reports, the present study found a single strong association on Mrg20 at 14.7 cM, although these GWAS were performed using single-gene models and complementarity would not be detectible using the historical phenotype scores. Future work using multi-genic models might prove fruitful.

Awn formation is associated with lemma color in certain genetic backgrounds (Matsuura, 1930; Ladizinsky, 1995). We were not able to demonstrate linkage between awn frequency and lemma color loci in these sub-samples, although weak evidence of association with lemma color (*p* = 0.002) was observed for marker BA_grs_c8269_158 on Mrg17 at 34 cM, just distal to the SNPs associated with awn frequency on that linkage group.

### Days to Anthesis and Growth Habit

Growth habit is evaluated in the NSGC by planting all genotypes in spring and identifying as winter type those which fail to flower or flower very late. The primary factor influencing outcome of the growth habit evaluation is presumed to be vernalization requirement, following from the premise that winter habit genotypes require vernalization to promote flowering whereas spring habit genotypes do not. However, vernalization response of oat evaluated in this way may be confounded with late flowering caused by photoperiod response and flowering time *per se* (rate of plant development), in particular since the vernalization requirement of oat is often facultative and weak (Brouwer and Flood, 1995). Both the SNP for growth habit on Mrg06 and that for days to anthesis on Mrg02 overlap with previously reported loci controlling traits related to fall/spring sown classification (Esvelt Klos et al., 2016).

In the present study, the significant marker for days to anthesis at 60.4cM on Mrg02 (GMI_ES03_c7453_413) in Aberdeen 83 co-locates with a second marginal association with growth habit in Aberdeen 96 and days to anthesis in Aberdeen 84 (GMI_ES_LB_2259, *p* = 6.16 × 10^-4^ and 1.96 × 10^-4^, respectively). This region contains heading date QTL regions mapped in the KO and OT populations (Siripoonwiwat et al., 1996; Holland et al., 1997, 2002); and lies between two QTL mapped by association in CORE elite oat germplasm at 30.1-34.1 cM and 71.6-85.2 cM, respectively (Esvelt Klos et al., 2016). Holland et al. (1997, 2002) measured heading date separately in both non-vernalized and vernalized plants and showed that the significant markers in this region did not affect vernalization response, supporting the hypothesis that the classification approach used by GRIN may confound vernalization response with flowering time *per se* (Supplementary Figure S3A).

An oat gene affecting flowering time, effective under short-day conditions and thought to be orthologous to flowering promoter genes *CONSTANS* in *Arabidopsis thaliana* and *Hd1* in rice, has been identified by Wight et al. (1994) as *Di1*. It was mapped by Locatelli et al. (2006) to Mrg02 and sequence homeology places it at either 34 or 81 cM, approximately (Supplementary Figure S3A).

On Mrg06, the statistically significant association with growth habit in the present study at 65.3 cM corresponds to a region of a weak linkage (*p* < 0.05) with heading date response to vernalization observed in the Kanota × Ogle population and with heading date after early planting in the field observed in the Ogle × TAM O-301 population (Holland et al., 1997, 2002; Supplementary Figure S3B). Additionally, SNPs on Mrg06 (at 45.2 and 67.6 cM) were associated with heading date in elite oat germplasm in 8 of 15 environments (Esvelt Klos et al., 2016). The apparent environment dependence of the effect of this QTL may be the explanation for failure to observe it in more than a single GRIN environment.

Numerous regions of the oat genome have been implicated in the control of flowering time, vernalization response, and photoperiod response (summarized in Esvelt Klos et al., 2016). In light of previous findings, the suggestive evidence of association with growth habit observed here on Mrg21 (*p* = 0.00005 for GMI_ES05_c8031_345 at 114.7 cM, and *p* = 0.0001 for GMI_ES02_c16331_464 at 122.8 cM) can be viewed with more confidence. However, we found no evidence of association with growth habit (or days to anthesis) on linkage groups Mrg09, Mrg12, or Mrg20, all of which have been reported to carry QTL mapped by both linkage and association and which carry candidate genes mapped by sequence homeology (Holland et al., 2002; Nava et al., 2012; Esvelt Klos et al., 2016; Supplementary Figure S3C; **Figure 4**).

### Traits for Which No Significant Markers were Identified

Significant associations were not identified in the present study for groat beta glucan, groat lipid, groat protein, plant height, test weight, lodging, yield, panicle length and density, smut reaction, straw breakage, shattering, greenbug reaction, and bundle weight. Previous studies have mapped QTL for groat beta glucan (Newell et al., 2012), groat lipids (Kianian et al., 1999; Zhu et al., 2004; Hizbai et al., 2012), and groat protein (Zhu et al., 2004; Hizbai et al., 2012; Tanhuanpää et al., 2012). Plant height has also been linked with multiple QTL in many studies. QTL conditioning yield, test weight and lodging have been identified in bi-parental populations studied by Siripoonwiwat et al. (1996) and De Koeyer et al. (2004), although QTL discovery in bi-parental populations can be more straightforward than in diverse panels due to the effects of allelic heterogeneity in the latter (Brachi et al., 2011). Failure of the present study to identify more QTL may be due to limitations on power arising from (i) study design in small, unreplicated groups, and (ii) relatively low marker density. The 2,588 markers used in the present study could be augmented in future studies by incorporating genotyping-by-sequencing to approach the 4,561 markers employed in the CORE GWAS (Esvelt Klos et al., 2016). In addition, many of the traits for which QTL were not identified are known to be complex, dependent on several component traits and subject to control by multiple QTL, some with context-dependent effects. These factors can be expected to complicate identification of QTL by GWAS.

## Conclusion

Cultivated oat is a widely adapted cereal with greater ecological range than either wheat or barley (Thomas, 1995). High morphological and genetic diversity make oats a valuable resource for plant breeding and agriculture, and the most judicious utilization of that resource relies on the growing body of literature clarifying our understanding of the crop’s population structure and genetic architecture.

The present study assembled a panel of historic and unimproved oat accessions from the NSGC with the goal of sampling germplasm that predates the influence of major breeding advances in the 20th century as a complement to similarly diverse but more recent panels. Population structure in this panel appeared to be stronger than that identified in previous studies. Primary explanatory variables were region of origin and lemma color. While ‘red’/‘common’ type or winter/spring growth habit classifications were relatively uninformative, available evidence was insufficient to establish whether they could be considered equivalent to the ‘fall sown’/‘spring sown’ classification found to account for structure in more recent oat panels.

Most accessions in the panel could be assigned to one of three population clusters. One of these clusters was more genetically distant from the other two and contained a disproportionately high concentration of accessions classified as ‘red’ type, winter, with red or gray lemmas and/or originating from Western Asia. This cluster appears to have been largely overlooked during twentieth century breeding and may be a valuable source of germplasm for future breeding work, particularly given its high average kernel weight.

Association mapping analyses conducted as part of the present study generated the first evidence of chromosomal regions controlling awn frequency, lemma color, kernels per spikelet and panicle type. Other evidence of association generally supported indications from previous studies. A single locus was associated with tolerance to BYD and may be orthologous to barley gene *Ryd3*. Six loci were associated with crown rust reaction, most of which appear to be linked with previously reported genes. Loci on Mrg01 and Mrg23 are reported here for the first time.

For future work, findings of the present study suggest the value of a more detailed approach to phenotyping and classification in cultivated oat. Key examples are the measurement of heat tolerance and cold tolerance, as well as the use experimental designs which separate tests of vernalization requirement, photoperiod response and flowering time *per se*. At the same time, genetic relationships between pairs of traits, such as plant height and lodging or yield and heading date, are imperfectly understood and will require well-designed experiments to resolve. More thorough phenotyping of this nature could greatly strengthen characterization of oat germplasm and explanations of population genetic diversity.

## Author Contributions

KEK initiated and organized the study; HB provided germplasm and phenotype information; SC performed genotyping work; LW, KEK, and JMB performed data analyses; LW drafted the manuscript, and KEK, JMB, and BAY contributed material; all authors read and approved the final manuscript.

## Conflict of Interest Statement

The authors declare that the research was conducted in the absence of any commercial or financial relationships that could be construed as a potential conflict of interest.

## Footnotes

1 http://www.ars-grin.gov

2 http://triticeaetoolbox.org/oat/

3 http://www.ars-grin.gov

4 http://unstats.un.org/unsd/methods/m49/m49regin.htm

## Abbreviations

6K: 6,000
BYD: Barley yellow dwarf
DArT: Diversity array technology
GRIN: Germplasm Resources Information Network of the U.S. Department of Agriculture
GWAS: Genome-wide association study
LD: Linkage disequilibrium
MAF: Minor allele frequency
Mrg: Merged linkage group
NSGC: National Small Grains Germplasm Collection of the U.S. Department of Agriculture
PCA: Principle component analysis
PCoA: Principle coordinate analysis
QTL: Quantitative trait locus
QTN: Quantitative trait nucleotide
RIL: Recombinant inbred line
SNP: Single nucleotide polymorphism
