# Supplementary material for: Population Structure and Genotype–Phenotype Associations in a Collection of Oat Landraces and Historic Cultivars
Source: Front Plant Sci. 2016 Jul 29;7:1077. doi: 10.3389/fpls.2016.01077 (PMC4965477; doi:10.3389/fpls.2016.01077)
Supplement: Supplementary file 1 [file Table_1.PDF]

Supplementary Material - Tables

Population structure and genotype-phenotype associations in a collection of oat landraces and historic cultivars

Louisa R. Winkler, J. Michael Bonman, Shiaoman Chao, Belayneh Admassu Yimer, Harold Bockelman and Kathy Esvelt-Klos*

* Correspondence: Kathy.klos@ars.usda.gov

Table S1.1 Qualitative phenotypes downloaded from GRIN for study/environments with information on 80 or more members of a 759-member panel of oat accessions.

Phenotype	Study/Environment	N	Distribution[a]
BYDV[b]	Urbana 84	439	0 3 8 17 21 31 72 240 47
	Urbana 85	208	0 0 1 6 9 14 51 115 12
Crown Rust Reaction[c]	Saint Paul 93	134	0 0 0 0 0 0 5 129
	Saint Paul 94	122	0 0 0 1 2 2 3 114
	Saint Paul 97	123	0 0 2 2 0 3 12 104
Growth habit[d]	Aberdeen 96	454	374 80
Lemma Color[e]	Aberdeen 11	675	48 11 177 416 23
Panicle type[f]	Aberdeen 83	118	109 1 8
	Aberdeen 84	111	108 1 2
	Aberdeen 85	304	279 6 19
Straw color[g]	Aberdeen 85	308	0 0 0 0 43 82 183

[a] The number of accessions in each category (from low to high or as otherwise specified) with spaces between categories. [b]Reaction to barley yellow dwarf virus where 1=resistant and 9=susceptible. [c]0=highly resistant and 9=susceptible. [d]Accessions scored as Spring or Winter. [e]1<46.00 (black), 4=46.01 to 57.99 (Grey), 6=58.00 to 71.99 (red), 9=72.00 to 82.99 (yellow), and 8>83.00 (amber/white). [f]1=equilateral, 5=intermediate, 9=unilateral. [g]1=black, 2=blue, 3=brown, 5=purple, 7=tan, 8=white/amber, and 9=yellow.

Table S1.2 Quantitative phenotypes downloaded from the Germplasm Resources Information Network (GRIN) from study/environments with information for >80 accessions in the present sample.

Phenotype	Study/Environment	N[a]	Mean	Standard Deviation
**Awn Frequency**	Aberdeen 83	118	2.47	2.72
	Aberdeen 84	109	2.78	2.19
	Aberdeen 85	305	3.17	1.96
**Beta Glucan[b]**	Madison 91	223	4.87	0.63
	Madison 95	303	4.31	0.69
**Bundle weight[c]**	Aberdeen 83	120	2.64	0.68
	Aberdeen 84	107	1.96	0.53
**Crown rust severity**	Saint Paul 93	137	60.84	16.09
	Saint Paul 94	121	58.58	20.89
	Saint Paul 97	123	56.93	13.18
**Greenbug reaction**	Stillwater 56	383	128.43	23.42
**Days to Anthesis**	Aberdeen 83	110	104.90	4.39
	Aberdeen 84	113	104.17	3.30
	Aberdeen 85	301	105.13	3.21
**Kernels per spikelet**	Aberdeen 85	309	2.11	0.31
**Lipid[b]**	Urbana	447	7.15	1.38
**Lodging**	Aberdeen 83	118	5.53	2.99
	Aberdeen 84	112	5.39	3.17
	Aberdeen 85	310	4.43	2.31
**Panicle Density**	Aberdeen 83	118	3.76	1.68
	Aberdeen 84	110	2.95	1.50
	Aberdeen 85	299	3.82	1.40
**Panicle length[d]**	Aberdeen 84	95	22.60	3.26
	Aberdeen 85	309	22.07	4.28
**Plant height[e]**	Aberdeen 83	117	126.50	12.84
	Aberdeen 84	110	108.45	11.33
	Aberdeen 85	311	104.24	17.60
**Protein[b]**	Madison 91	223	18.37	1.85
	Madison 95	303	19.74	1.26
**Shattering**	Aberdeen 85	309	6.16	1.85
**Smut[f]**	Saint Paul 93	142	54.76	37.78
	Saint Paul 94	119	34.09	40.15
**Spikelets per panicle**	Aberdeen 83	125	30.26	8.11
	Aberdeen 84	95	35.16	11.99
	Aberdeen 85	309	32.30	12.50
**Straw breakage**	Aberdeen 85	309	3.08	1.38
**Test Weight[g]**	Aberdeen 83	113	44.65	4.32
	Aberdeen 84	115	47.68	4.61
	Aberdeen 85	310	47.55	3.41
**Yield[h]**	Aberdeen 83	114	285.34	112.81
	Aberdeen 84	115	298.56	97.93
	Aberdeen 85	310	286.95	73.89

[a]The number of accessions in the present study with GRIN observations. [b]Content of the kernel as the percent dry weight. [c]Pounds per plot. [d]Average distance (cm) from lowest node to tip of apical spike. [e]Average distance (cm) from the ground to the panicle tops. [f]Percent of the susceptible check. [g]Kilograms per hectoliter of grain. [h]The weight of harvested grain in grams per un-replicated 2.44 meter row.
