# Supplementary material for: Population Structure and Genotype–Phenotype Associations in a Collection of Oat Landraces and Historic Cultivars
Source: Front Plant Sci. 2016 Jul 29;7:1077. doi: 10.3389/fpls.2016.01077 (PMC4965477; doi:10.3389/fpls.2016.01077)
Supplement: Supplementary file 2 [file Image_1.PDF]

Supplementary Material - Figures

Population structure and genotype-phenotype associations in a collection of oat landraces and historic cultivars

Louisa R. Winkler, J. Michael Bonman, Shiaoman Chao, Belayneh Admassu Yimer, Harold Bockelman and Kathy Esvelt-Klos*

* Correspondence: Kathy.klos@ars.usda.gov

Figure S1 Comparison of a 759-member panel of historic and unimproved accessions ('Diversity Sample') included in the present study and the NSGC *Avena sativa* collection with respect to (**A**) improvement status and collection year, and (**B**) improvement status and collection region.

S1(A)

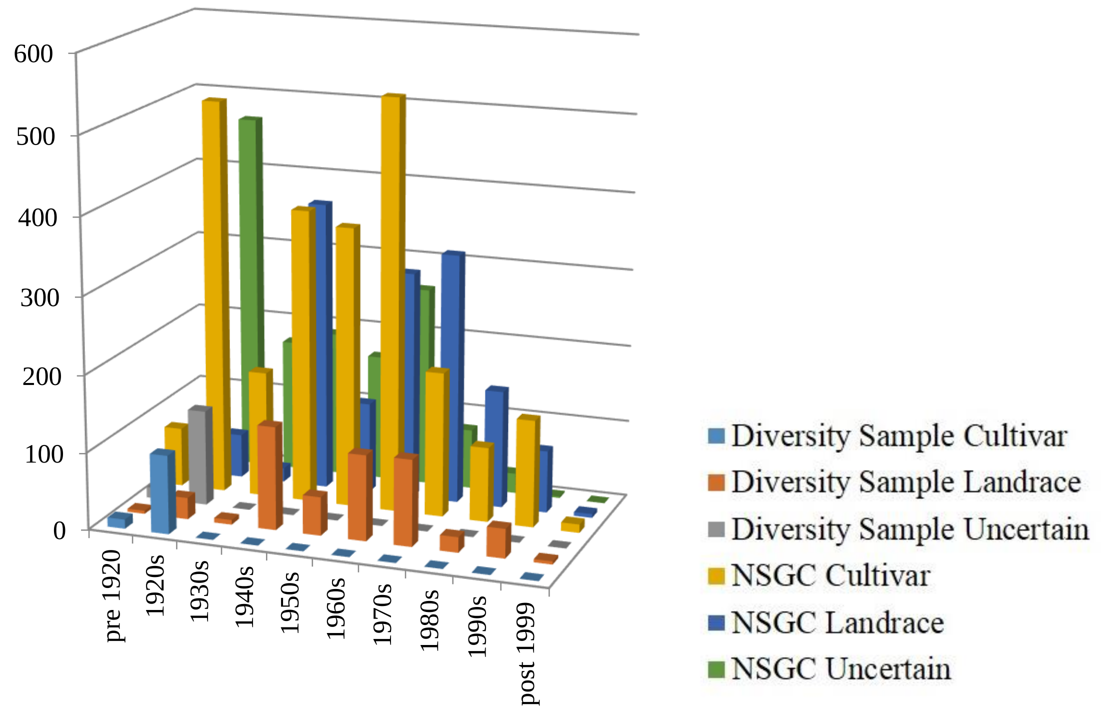

S1(B)

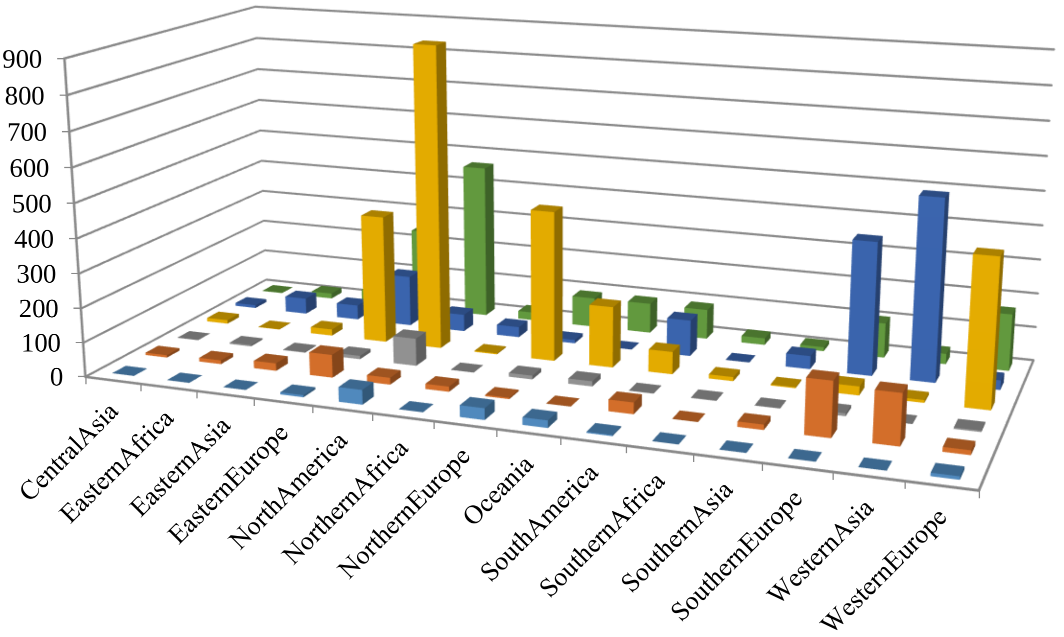

Figure S2. Left: Manhattan plots showing marker-trait associations from a genome-wide association study using a panel of 759 oat landraces and historic cultivars genotyped for 2,588 polymorphic SNPs with map positions, together with phenotype information from GRIN. Y-axis shows $-\log_{10}$ of *p*-values calculated in the association model; points are colored according to chromosome assignment (Mrg) in the oat consensus map; dotted blue line shows significance threshold ($p=1.93\times10^{-5}$). Right: Quantile-quantile plots showing *p*-values calculated in the association model (y-axis) plotted against the null distribution (x); all values transformed by $-\log_{10}$.

S2(A)

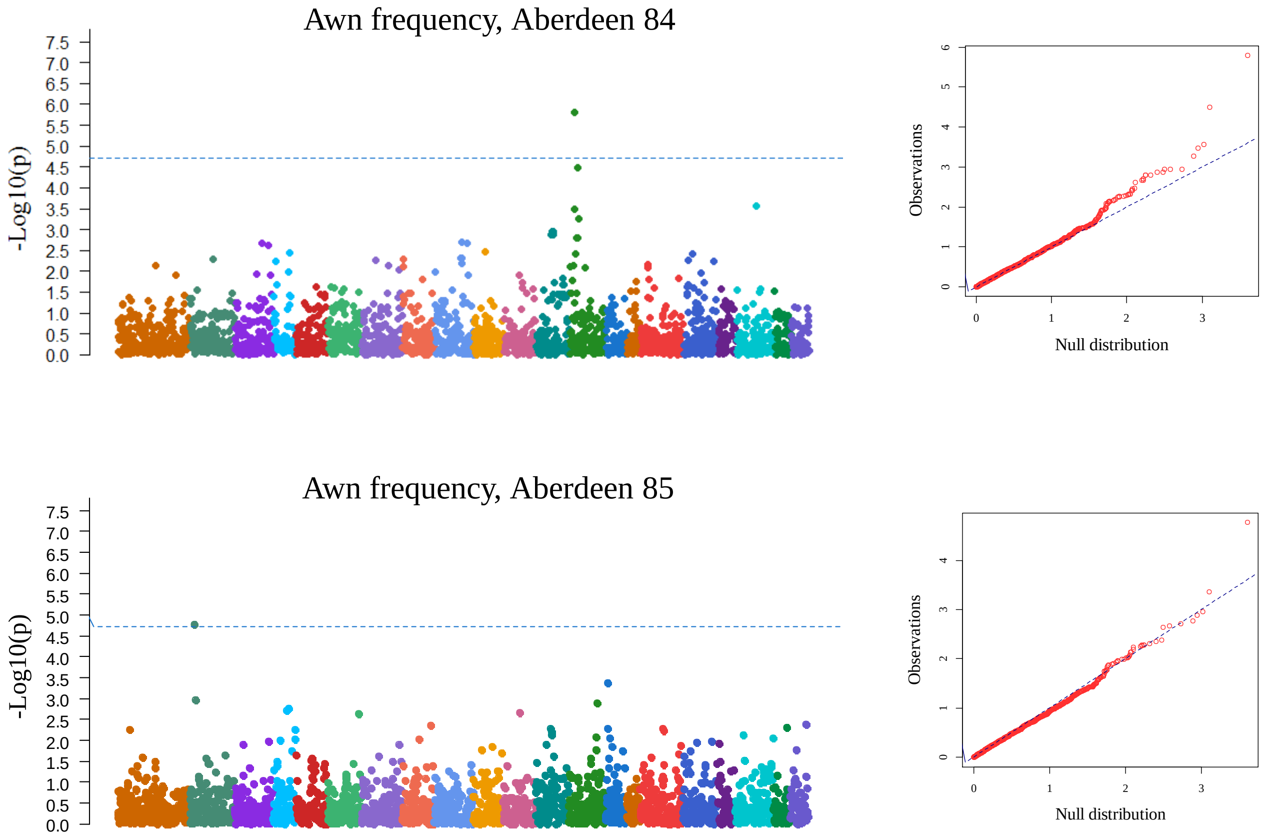

S2(B)

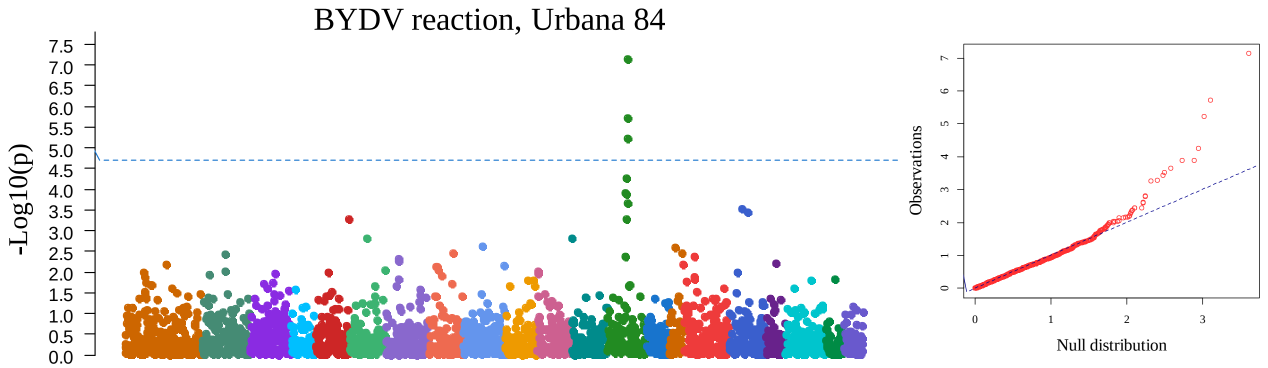

S2(C)

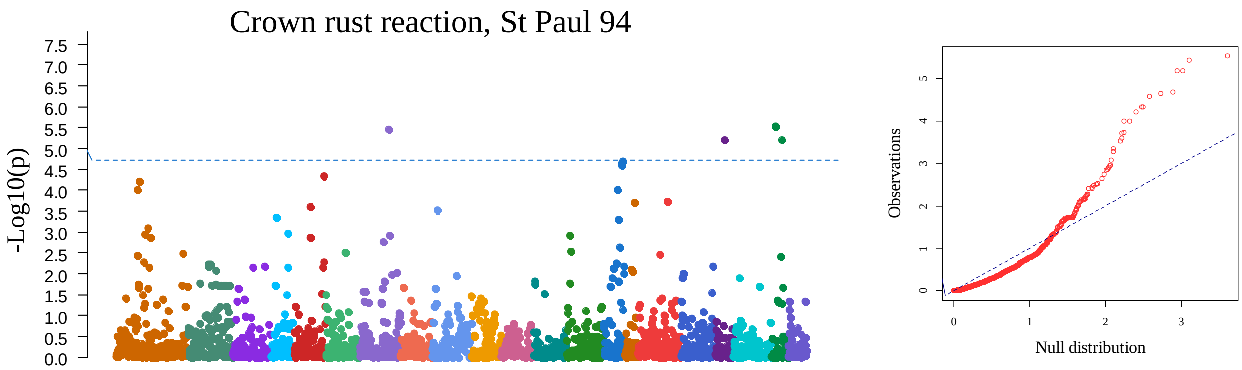
Crown rust reaction, St Paul 94
-Log10(p)
0.0 0.5 1.0 1.5 2.0 2.5 3.0 3.5 4.0 4.5 5.0 5.5 6.0 6.5 7.0 7.5
Observations
0 1 2 3 4 5
Null distribution
0 1 2 3

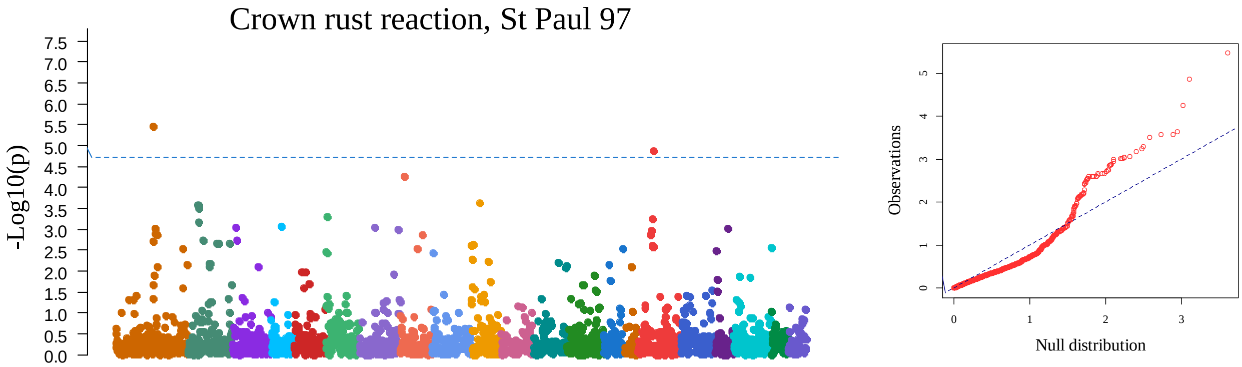
Crown rust reaction, St Paul 97
-Log10(p)
0.0 0.5 1.0 1.5 2.0 2.5 3.0 3.5 4.0 4.5 5.0 5.5 6.0 6.5 7.0 7.5
Observations
0 1 2 3 4 5
Null distribution
0 1 2 3

S2(D)

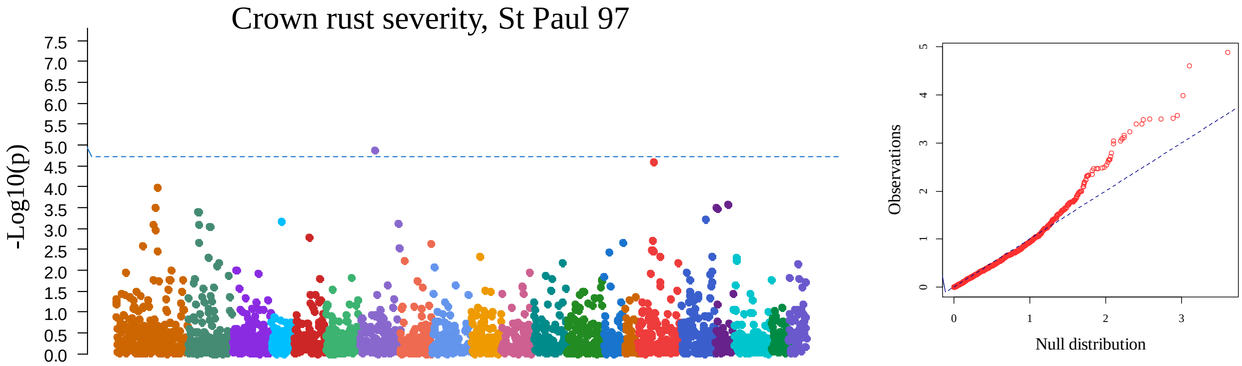
Crown rust severity, St Paul 97
-Log10(p)
0.0 0.5 1.0 1.5 2.0 2.5 3.0 3.5 4.0 4.5 5.0 5.5 6.0 6.5 7.0 7.5
Observations
0 1 2 3 4 5
Null distribution
0 1 2 3

S2(E)

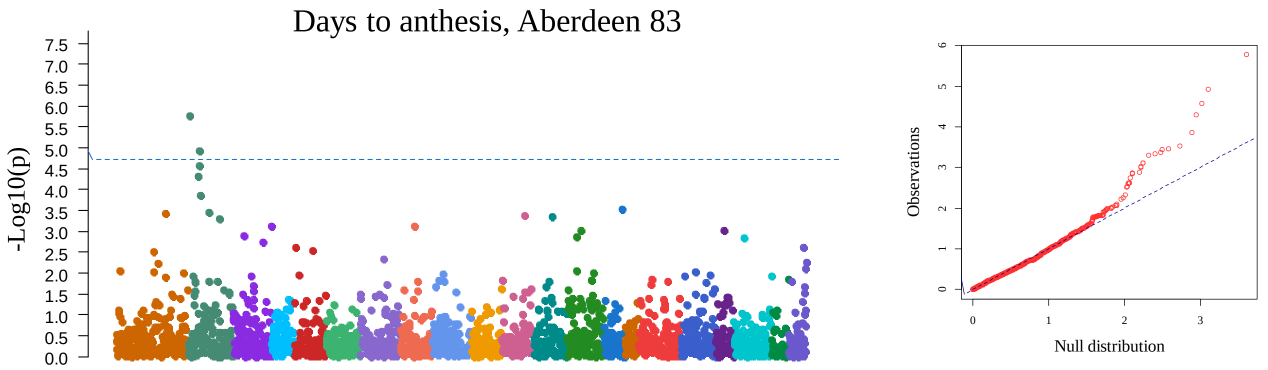

S2(F)

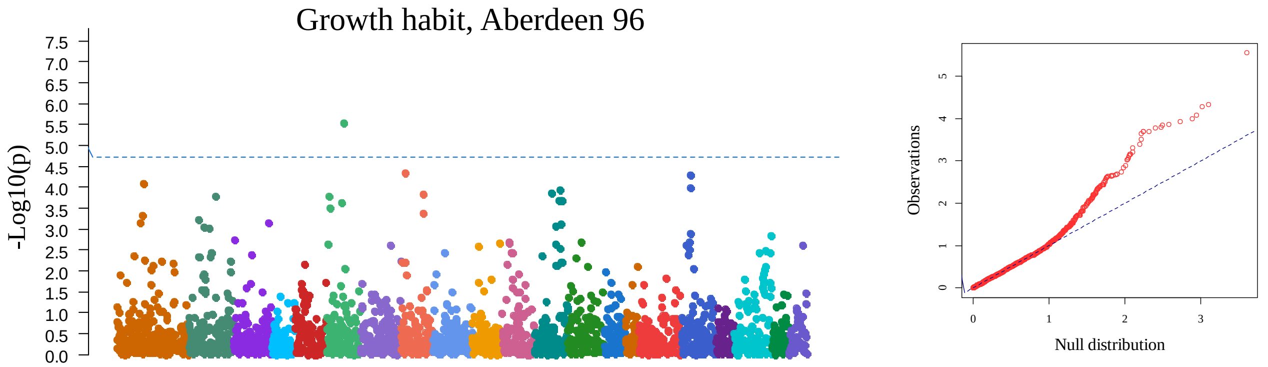

S2(G)

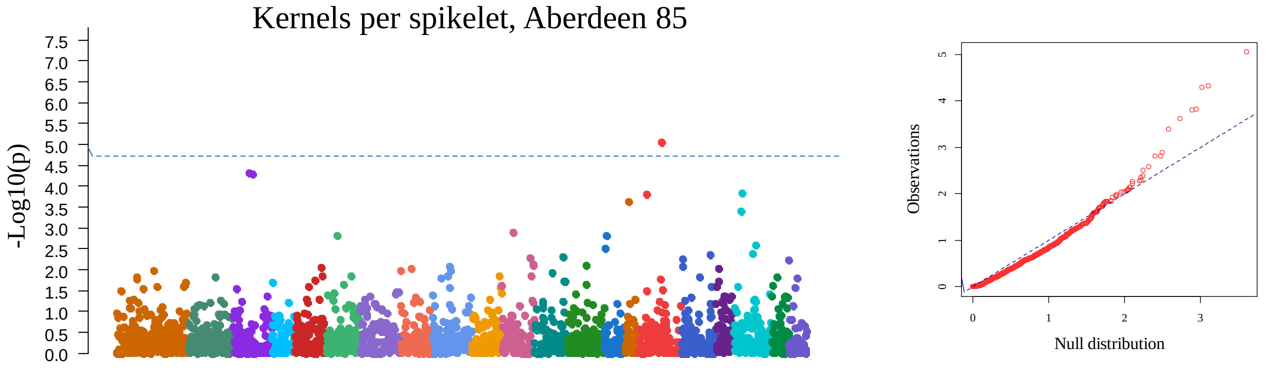

S2(H)

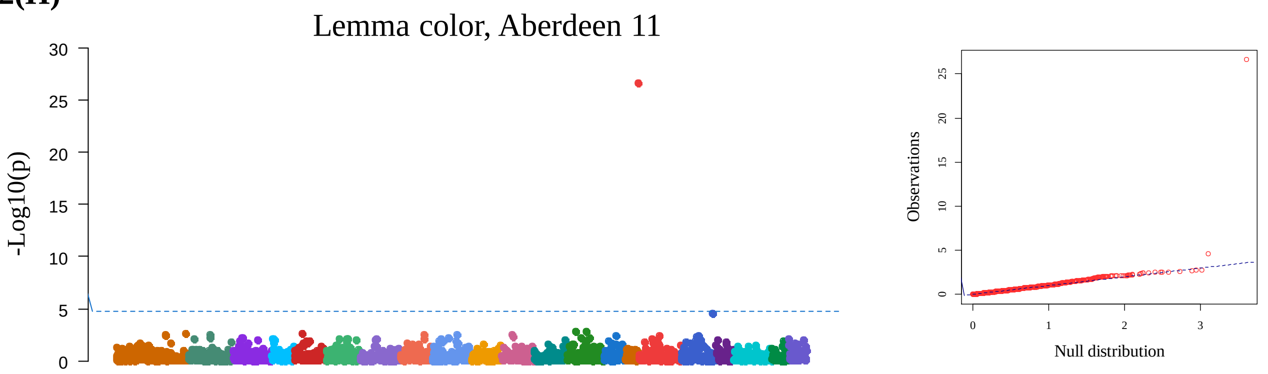

S2(I)

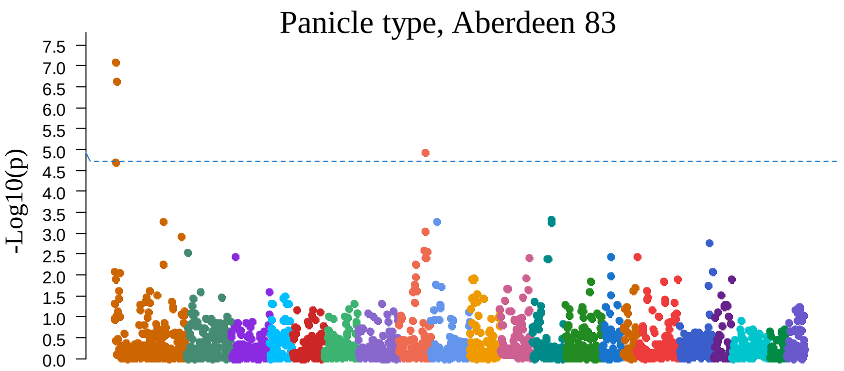
Panicle type, Aberdeen 83
-Log10(p)
7.5
7.0
6.5
6.0
5.5
5.0
4.5
4.0
3.5
3.0
2.5
2.0
1.5
1.0
0.5
0.0

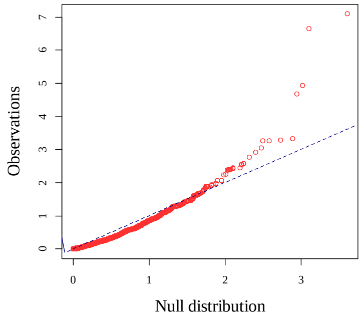
Observations
Null distribution
0
1
2
3
4
5
6
7

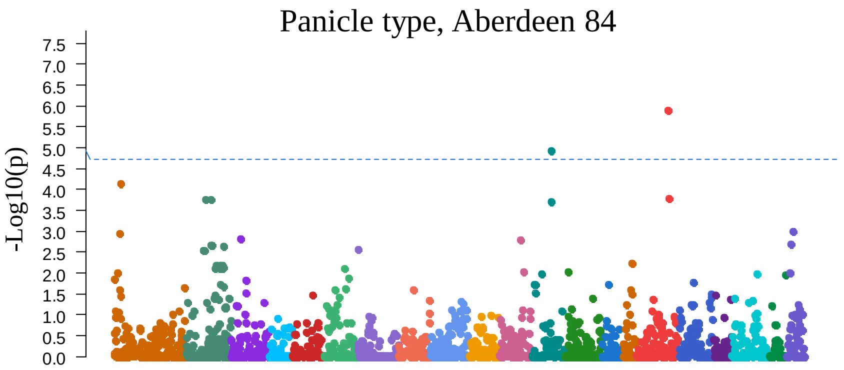
Panicle type, Aberdeen 84
-Log10(p)
7.5
7.0
6.5
6.0
5.5
5.0
4.5
4.0
3.5
3.0
2.5
2.0
1.5
1.0
0.5
0.0

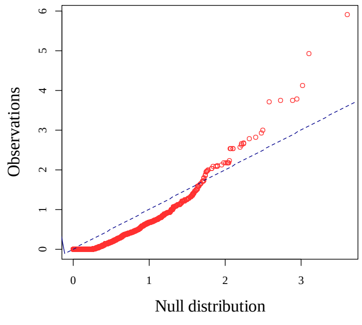
Observations
Null distribution
0
1
2
3
4
5
6

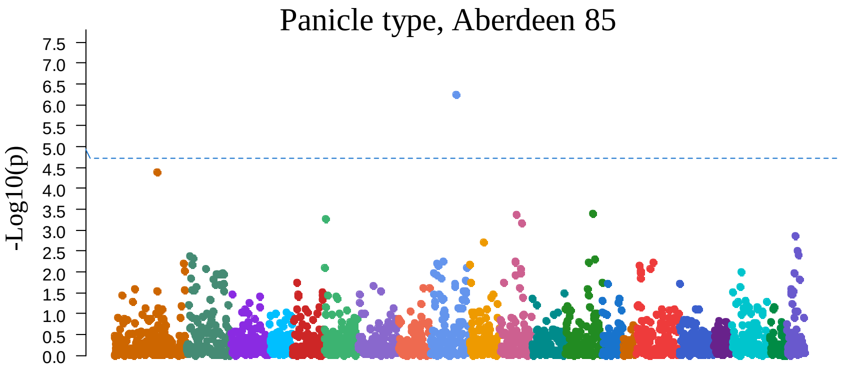
Panicle type, Aberdeen 85
-Log10(p)
7.5
7.0
6.5
6.0
5.5
5.0
4.5
4.0
3.5
3.0
2.5
2.0
1.5
1.0
0.5
0.0

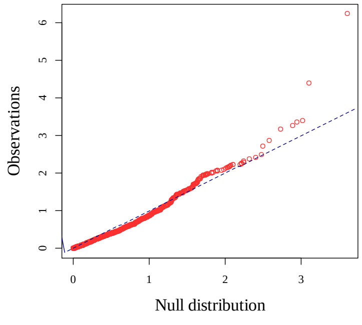
Observations
Null distribution
0
1
2
3
4
5
6

Fig. S3(A)-S3(F) Oat linkage groups showing marker-trait associations identified in the present study alongside those identified in previous work (see main text Fig.4 for Mrg17 and 20).[a]

S3(A)

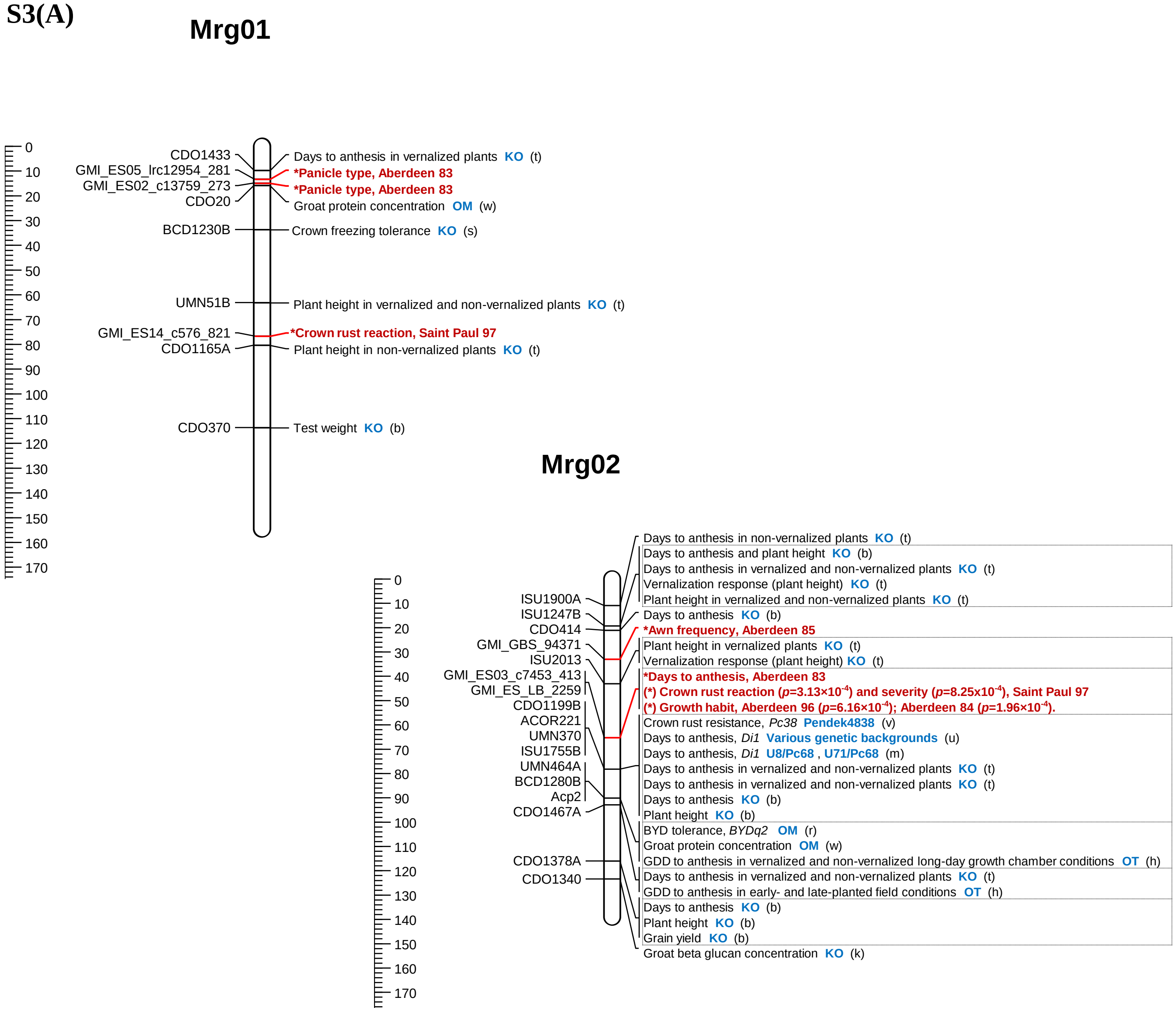

[a]Results from the present study are in bold red font, and the environment(s) in which an association was found is recorded. Those marked with * were significant at a threshold of $p<1.93x10^{-5}$, and those marked with (*) were near-significant at a threshold of $4.0x10^{-5}$ unless otherwise stated. Where previous findings are reported, a designation of the population in which the study was conducted is displayed in blue font, and the reference is given by a lower-case letter in brackets (see Fig. 4 in main text for key to references and population abbreviations). Putative genes are in italics. Where a trait association was mapped to an interval rather than a single marker, the figure displays the marker which showed the strongest association and/or was mapped the closest. Boxes are drawn around associations mapping to the same locus. Stem rust is caused by *Puccinia graminis* f. sp. *avenae*. crown rust by *Puccinia coronata* f. sp. *avenae*. GDD stands for growing degree days. See supplementary figures S3A-R for remaining linkage groups. The figure was constructed with the help of MapChart v2.3 (Voorrips, 2002).

S3(B)

Mrg03

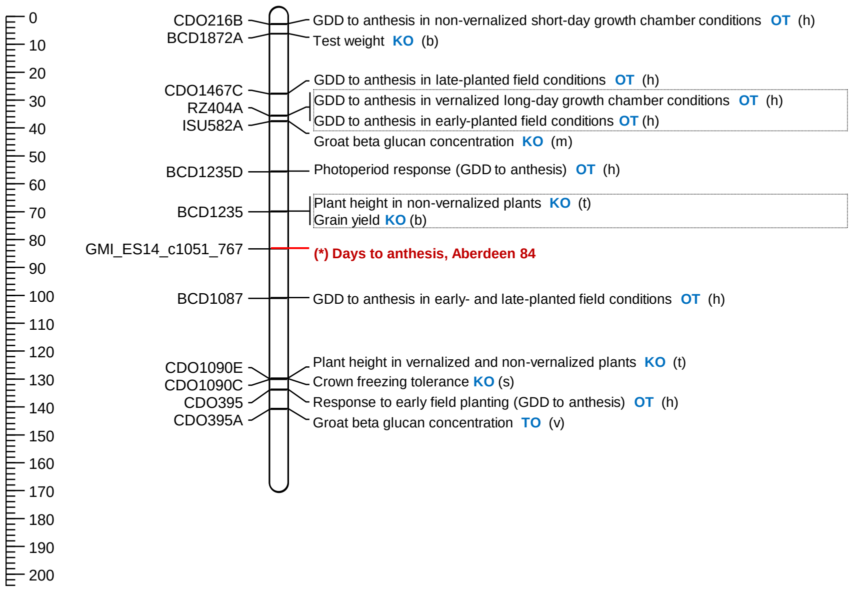

Mrg04

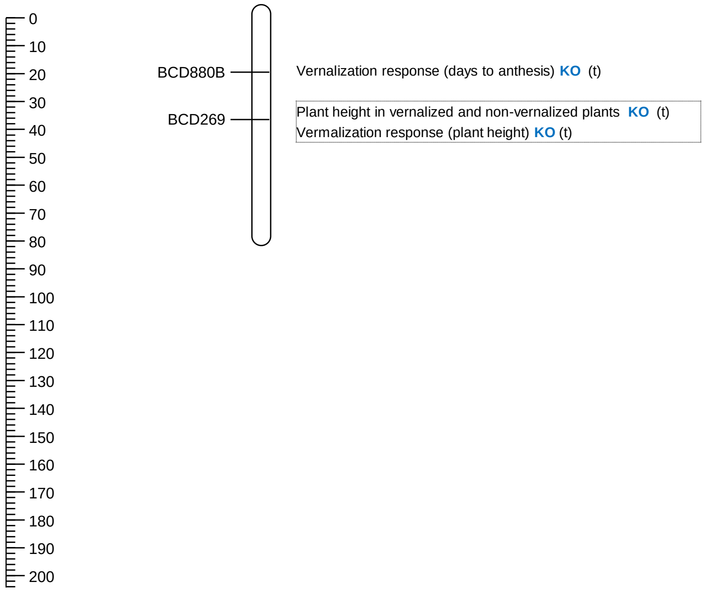

S3(C)

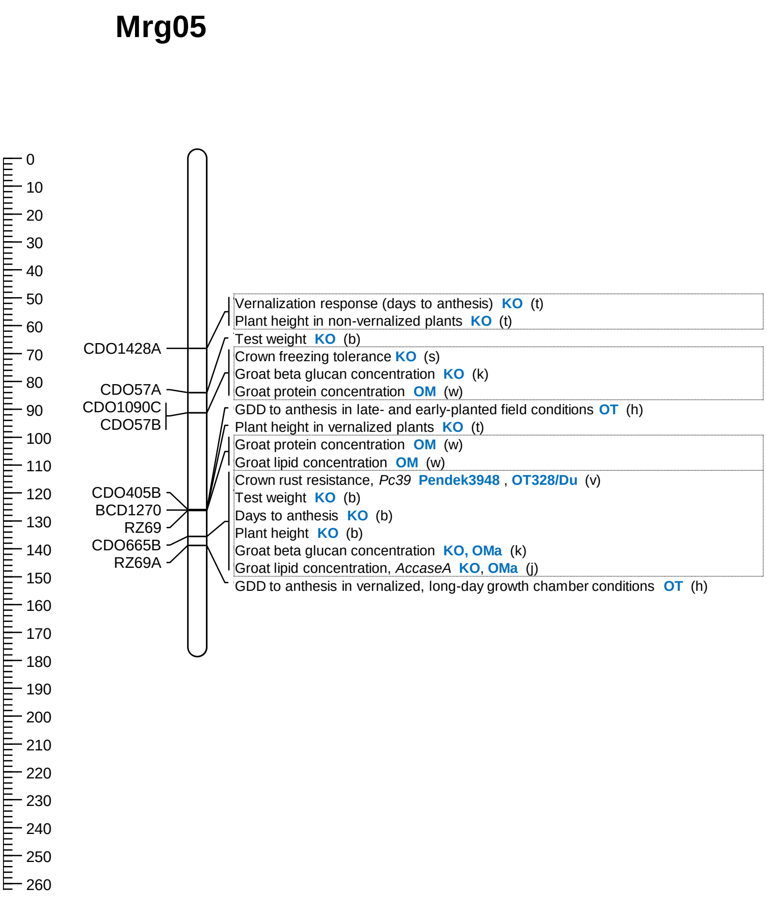

Mrg05
0
10
20
30
40
50
60
70
80
90
100
110
120
130
140
150
160
170
180
190
200
210
220
230
240
250
260
CDO1428A
CDO57A
CDO1090C
CDO57B
CDO405B
BCD1270
RZ69
CDO665B
RZ69A
Vernalization response (days to anthesis) KO (t)
Plant height in non-vernalized plants KO (t)
Test weight KO (b)
Crown freezing tolerance KO (s)
Groat beta glucan concentration KO (k)
Groat protein concentration OM (w)
GDD to anthesis in late- and early-planted field conditions OT (h)
Plant height in vernalized plants KO (t)
Groat protein concentration OM (w)
Groat lipid concentration OM (w)
Crown rust resistance, Pc39 Pendek3948 , OT328/Du (v)
Test weight KO (b)
Days to anthesis KO (b)
Plant height KO (b)
Groat beta glucan concentration KO, OMa (k)
Groat lipid concentration, AccaseA KO, OMa (j)
GDD to anthesis in vernalized, long-day growth chamber conditions OT (h)

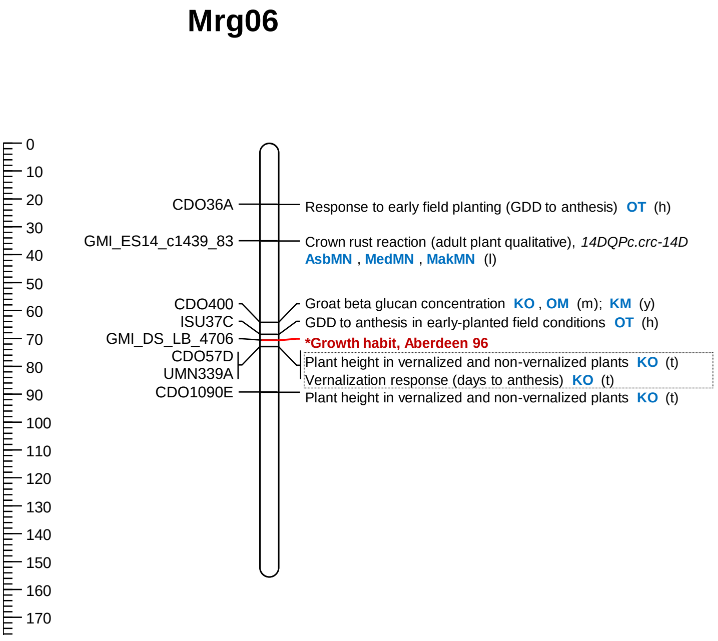

Mrg06
0
10
20
30
40
50
60
70
80
90
100
110
120
130
140
150
160
170
CDO36A
GMI_ES14_c1439_83
CDO400
ISU37C
GMI_DS_LB_4706
CDO57D
UMN339A
CDO1090E
Response to early field planting (GDD to anthesis) OT (h)
Crown rust reaction (adult plant qualitative), 14DQPc.crc-14D
AsbMN , MedMN , MakMN (l)
Groat beta glucan concentration KO , OM (m); KM (y)
GDD to anthesis in early-planted field conditions OT (h)
*Growth habit, Aberdeen 96
Plant height in vernalized and non-vernalized plants KO (t)
Vernalization response (days to anthesis) KO (t)
Plant height in vernalized and non-vernalized plants KO (t)

S3(D)

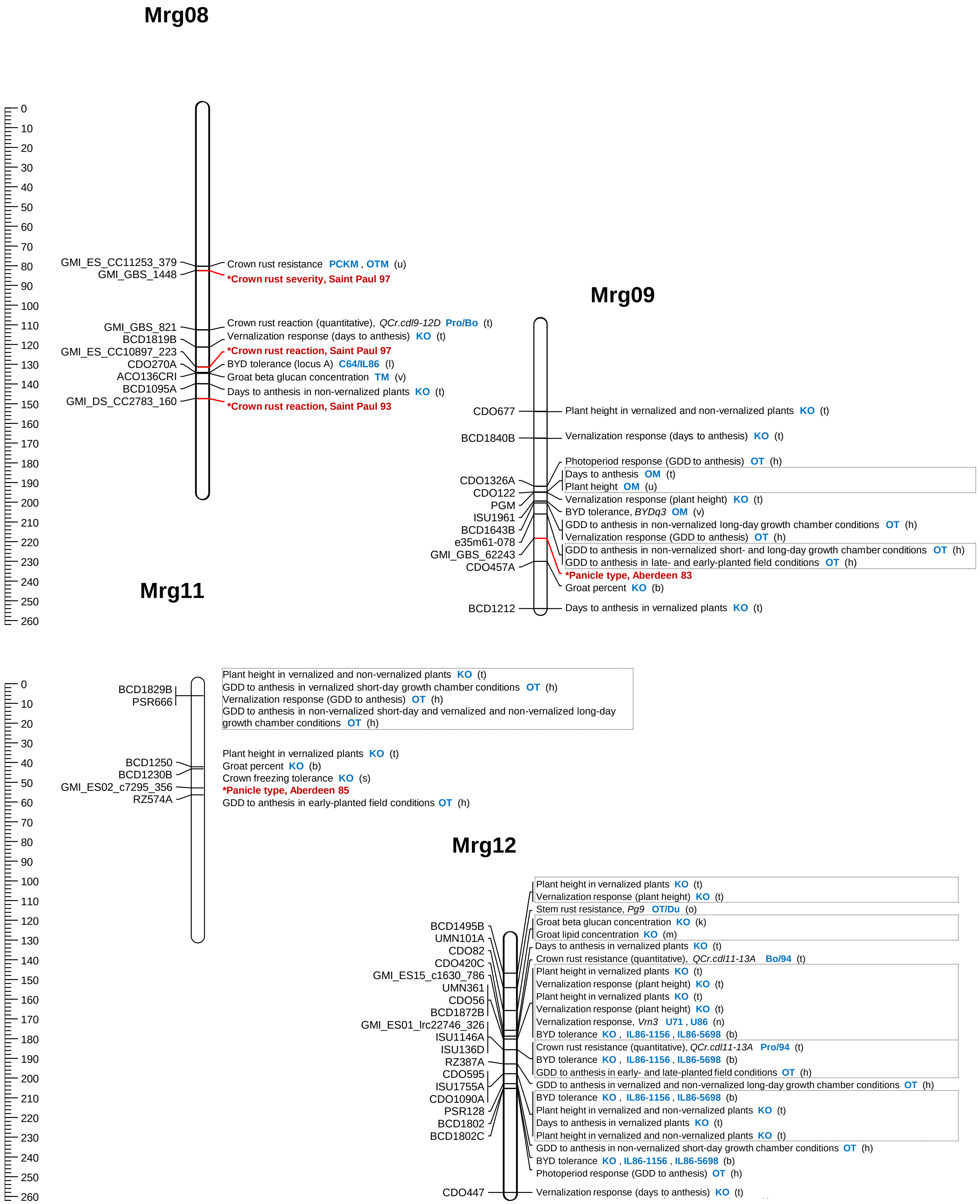

Mrg08
0
10
20
30
40
50
60
70
80
90
100
110
120
130
140
150
160
170
180
190
200
210
220
230
240
250
260
GMI_ES_CC11253_379
GMI_GBS_1448
GMI_GBS_821
BCD1819B
GMI_ES_CC10897_223
CDO270A
ACO136CRI
BCD1095A
GMI_DS_CC2783_160
Crown rust resistance PCKM , OTM (u)
*Crown rust severity, Saint Paul 97
Crown rust reaction (quantitative), QCr.cdl9-12D Pro/Bo (t)
Vernalization response (days to anthesis) KO (t)
*Crown rust reaction, Saint Paul 97
BYD tolerance (locus A) C64/IL86 (l)
Groat beta glucan concentration TM (v)
Days to anthesis in non-vernalized plants KO (t)
*Crown rust reaction, Saint Paul 93
Mrg09
CDO677
BCD1840B
CDO1326A
CDO122
PGM
ISU1961
BCD1643B
e35m61-078
GMI_GBS_62243
CDO457A
BCD1212
Plant height in vernalized and non-vernalized plants KO (t)
Vernalization response (days to anthesis) KO (t)
Photoperiod response (GDD to anthesis) OT (h)
Days to anthesis OM (t)
Plant height OM (u)
Vernalization response (plant height) KO (t)
BYD tolerance, BYDq3 OM (v)
GDD to anthesis in non-vernalized long-day growth chamber conditions OT (h)
Vernalization response (GDD to anthesis) OT (h)
GDD to anthesis in non-vernalized short- and long-day growth chamber conditions OT (h)
GDD to anthesis in late- and early-planted field conditions OT (h)
*Panicle type, Aberdeen 83
Groat percent KO (b)
Days to anthesis in vernalized plants KO (t)
Mrg11
0
10
20
30
40
50
60
70
80
90
100
110
120
130
140
150
160
170
180
190
200
210
220
230
240
250
260
BCD1829B
PSR666
BCD1250
BCD1230B
GMI_ES02_c7295_356
RZ574A
Plant height in vernalized and non-vernalized plants KO (t)
GDD to anthesis in vernalized short-day growth chamber conditions OT (h)
Vernalization response (GDD to anthesis) OT (h)
GDD to anthesis in non-vernalized short-day and vernalized and non-vernalized long-day growth chamber conditions OT (h)
Plant height in vernalized plants KO (t)
Groat percent KO (b)
Crown freezing tolerance KO (s)
*Panicle type, Aberdeen 85
GDD to anthesis in early-planted field conditions OT (h)
Mrg12
BCD1495B
UMN101A
CDO82
CDO420C
GMI_ES15_c1630_786
UMN361
CDO56
BCD1872B
GMI_ES01_lrc22746_326
ISU1146A
ISU136D
RZ387A
CDO595
ISU1755A
CDO1090A
PSR128
BCD1802
BCD1802C
CDO447
Plant height in vernalized plants KO (t)
Vernalization response (plant height) KO (t)
Stem rust resistance, Pg9 OT/Du (o)
Groat beta glucan concentration KO (k)
Groat lipid concentration KO (m)
Days to anthesis in vernalized plants KO (t)
Crown rust resistance (quantitative), QCr.cdl11-13A Bo/94 (t)
Plant height in vernalized plants KO (t)
Vernalization response (plant height) KO (t)
Plant height in vernalized plants KO (t)
Vernalization response (plant height) KO (t)
Vernalization response, Vrn3 U71 , U86 (n)
BYD tolerance KO , IL86-1156 , IL86-5698 (b)
Crown rust resistance (quantitative), QCr.cdl11-13A Pro/94 (t)
BYD tolerance KO , IL86-1156 , IL86-5698 (b)
GDD to anthesis in early- and late-planted field conditions OT (h)
GDD to anthesis in vernalized and non-vernalized long-day growth chamber conditions OT (h)
BYD tolerance KO , IL86-1156 , IL86-5698 (b)
Plant height in vernalized and non-vernalized plants KO (t)
Days to anthesis in vernalized plants KO (t)
Plant height in vernalized and non-vernalized plants KO (t)
GDD to anthesis in non-vernalized short-day growth chamber conditions OT (h)
BYD tolerance KO , IL86-1156 , IL86-5698 (b)
Photoperiod response (GDD to anthesis) OT (h)
Vernalization response (days to anthesis) KO (t)

S3(E)

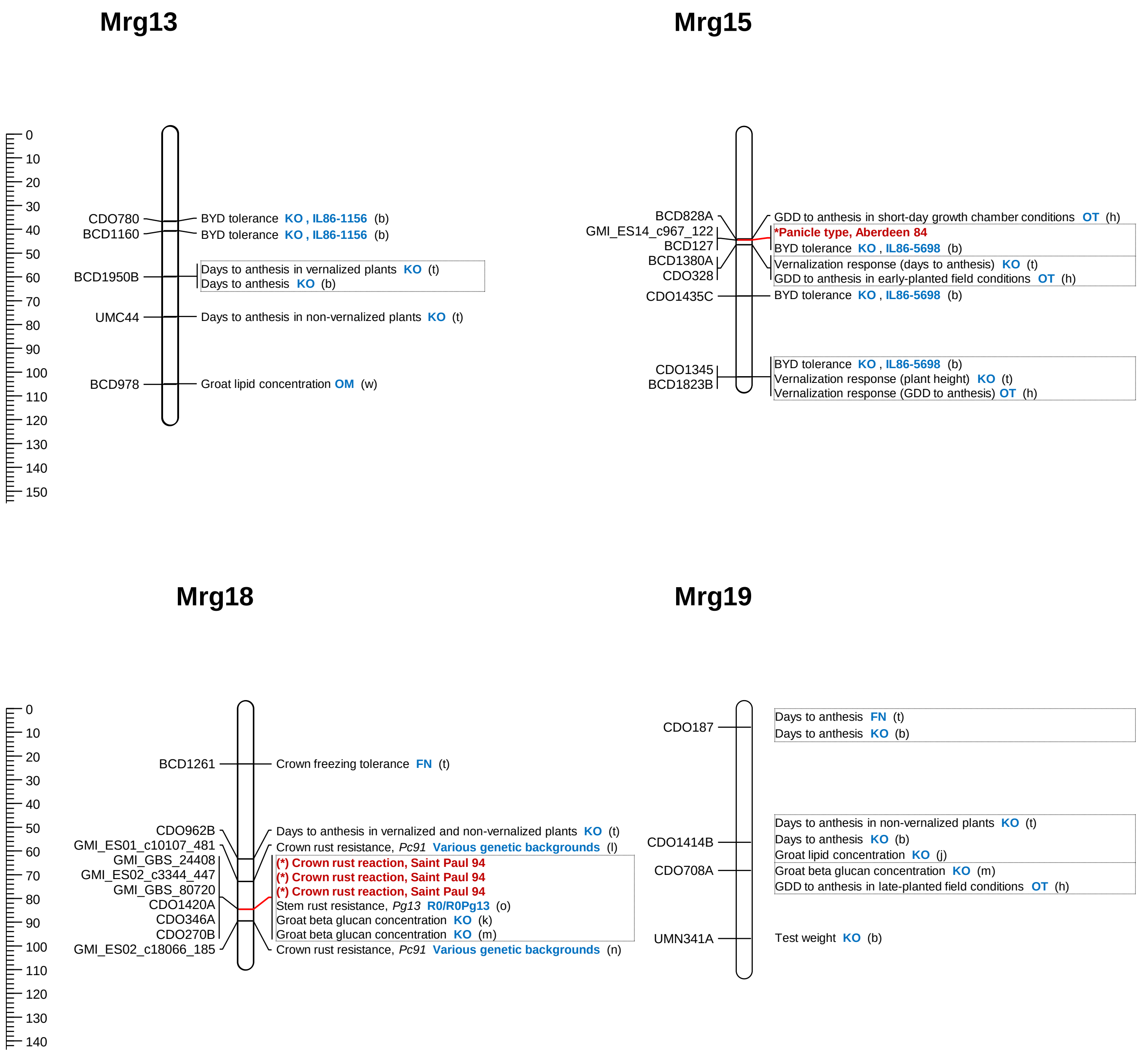

Mrg13
Mrg15
0
10
20
30
40
50
60
70
80
90
100
110
120
130
140
150
CDO780
BCD1160
BCD1950B
UMC44
BCD978
BYD tolerance KO , IL86-1156 (b)
BYD tolerance KO , IL86-1156 (b)
Days to anthesis in vernalized plants KO (t)
Days to anthesis KO (b)
Days to anthesis in non-vernalized plants KO (t)
Groat lipid concentration OM (w)
BCD828A
GMI_ES14_c967_122
BCD127
BCD1380A
CDO328
CDO1435C
CDO1345
BCD1823B
GDD to anthesis in short-day growth chamber conditions OT (h)
*Panicle type, Aberdeen 84
BYD tolerance KO , IL86-5698 (b)
Vernalization response (days to anthesis) KO (t)
GDD to anthesis in early-planted field conditions OT (h)
BYD tolerance KO , IL86-5698 (b)
BYD tolerance KO , IL86-5698 (b)
Vernalization response (plant height) KO (t)
Vernalization response (GDD to anthesis) OT (h)
Mrg18
Mrg19
BCD1261
CDO962B
GMI_ES01_c10107_481
GMI_GBS_24408
GMI_ES02_c3344_447
GMI_GBS_80720
CDO1420A
CDO346A
CDO270B
GMI_ES02_c18066_185
Crown freezing tolerance FN (t)
Days to anthesis in vernalized and non-vernalized plants KO (t)
Crown rust resistance, Pc91 Various genetic backgrounds (l)
(*) Crown rust reaction, Saint Paul 94
(*) Crown rust reaction, Saint Paul 94
(*) Crown rust reaction, Saint Paul 94
Stem rust resistance, Pg13 R0/R0Pg13 (o)
Groat beta glucan concentration KO (k)
Groat beta glucan concentration KO (m)
Crown rust resistance, Pc91 Various genetic backgrounds (n)
CDO187
CDO1414B
CDO708A
UMN341A
Days to anthesis FN (t)
Days to anthesis KO (b)
Days to anthesis in non-vernalized plants KO (t)
Days to anthesis KO (b)
Groat lipid concentration KO (j)
Groat beta glucan concentration KO (m)
GDD to anthesis in late-planted field conditions OT (h)
Test weight KO (b)

S3(F)

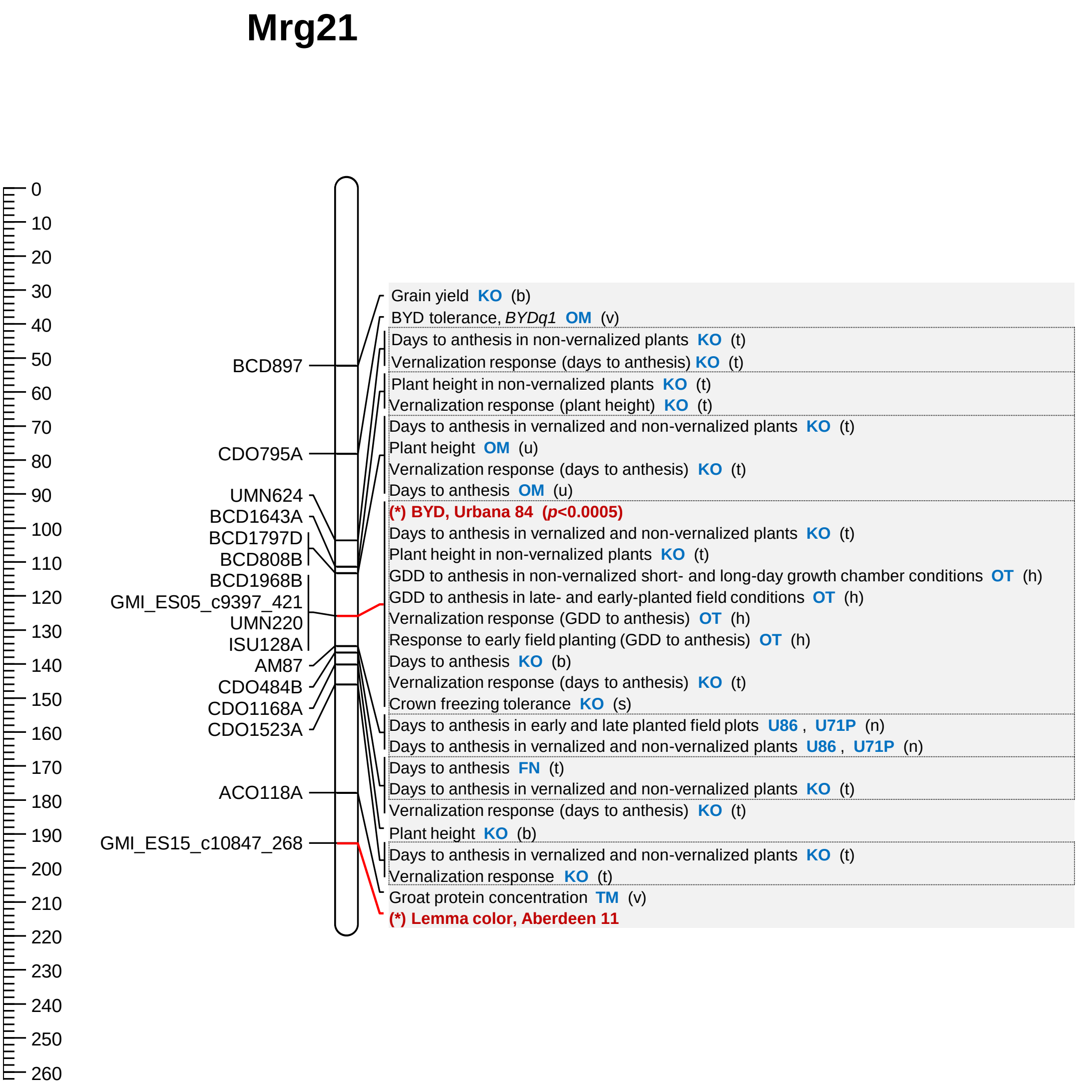

Mrg21
BCD897
CDO795A
UMN624
BCD1643A
BCD1797D
BCD808B
BCD1968B
GMI_ES05_c9397_421
UMN220
ISU128A
AM87
CDO484B
CDO1168A
CDO1523A
ACO118A
GMI_ES15_c10847_268
Grain yield KO (b)
BYD tolerance, BYDq1 OM (v)
Days to anthesis in non-vernalized plants KO (t)
Vernalization response (days to anthesis) KO (t)
Plant height in non-vernalized plants KO (t)
Vernalization response (plant height) KO (t)
Days to anthesis in vernalized and non-vernalized plants KO (t)
Plant height OM (u)
Vernalization response (days to anthesis) KO (t)
Days to anthesis OM (u)
(*) BYD, Urbana 84 (p<0.0005)
Days to anthesis in vernalized and non-vernalized plants KO (t)
Plant height in non-vernalized plants KO (t)
GDD to anthesis in non-vernalized short- and long-day growth chamber conditions OT (h)
GDD to anthesis in late- and early-planted field conditions OT (h)
Vernalization response (GDD to anthesis) OT (h)
Response to early field planting (GDD to anthesis) OT (h)
Days to anthesis KO (b)
Vernalization response (days to anthesis) KO (t)
Crown freezing tolerance KO (s)
Days to anthesis in early and late planted field plots U86 , U71P (n)
Days to anthesis in vernalized and non-vernalized plants U86 , U71P (n)
Days to anthesis FN (t)
Days to anthesis in vernalized and non-vernalized plants KO (t)
Vernalization response (days to anthesis) KO (t)
Plant height KO (b)
Days to anthesis in vernalized and non-vernalized plants KO (t)
Vernalization response KO (t)
Groat protein concentration TM (v)
(*) Lemma color, Aberdeen 11

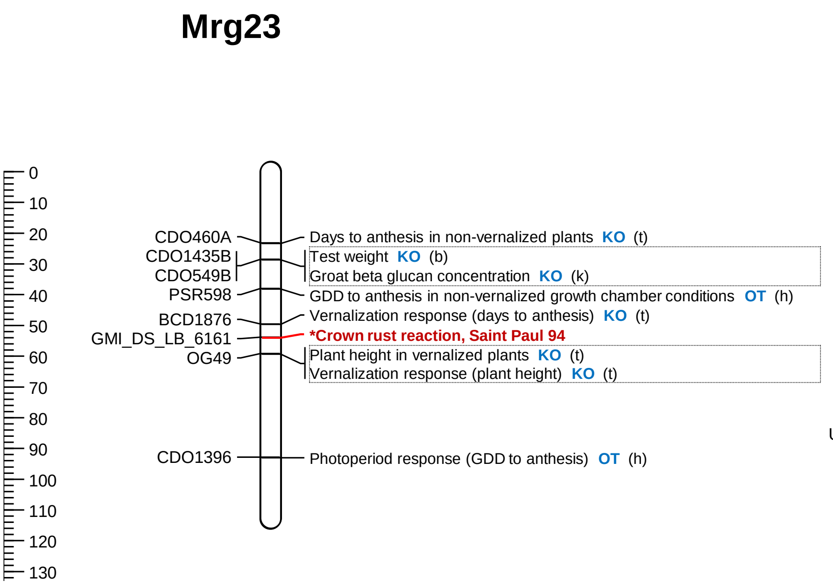

Mrg23
CDO460A
CDO1435B
CDO549B
PSR598
BCD1876
GMI_DS_LB_6161
OG49
CDO1396
Days to anthesis in non-vernalized plants KO (t)
Test weight KO (b)
Groat beta glucan concentration KO (k)
GDD to anthesis in non-vernalized growth chamber conditions OT (h)
Vernalization response (days to anthesis) KO (t)
*Crown rust reaction, Saint Paul 94
Plant height in vernalized plants KO (t)
Vernalization response (plant height) KO (t)
Photoperiod response (GDD to anthesis) OT (h)

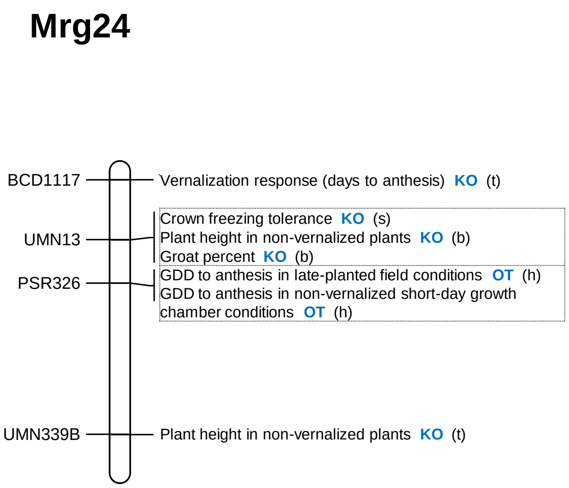

Mrg24
BCD1117
UMN13
PSR326
UMN339B
Vernalization response (days to anthesis) KO (t)
Crown freezing tolerance KO (s)
Plant height in non-vernalized plants KO (b)
Groat percent KO (b)
GDD to anthesis in late-planted field conditions OT (h)
GDD to anthesis in non-vernalized short-day growth chamber conditions OT (h)
Plant height in non-vernalized plants KO (t)

S3(G)

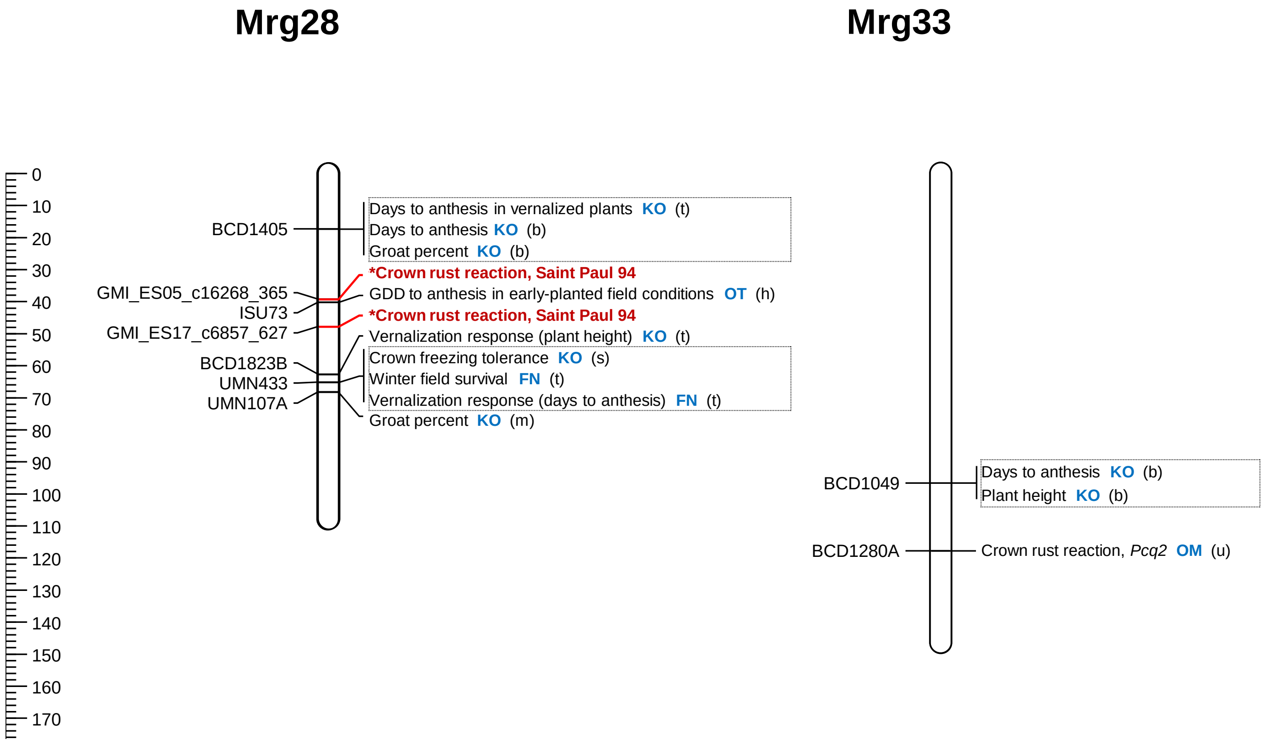

Mrg28
Mrg33
0
10
20
30
40
50
60
70
80
90
100
110
120
130
140
150
160
170
BCD1405
GMI_ES05_c16268_365
ISU73
GMI_ES17_c6857_627
BCD1823B
UMN433
UMN107A
Days to anthesis in vernalized plants KO (t)
Days to anthesis KO (b)
Groat percent KO (b)
*Crown rust reaction, Saint Paul 94
GDD to anthesis in early-planted field conditions OT (h)
*Crown rust reaction, Saint Paul 94
Vernalization response (plant height) KO (t)
Crown freezing tolerance KO (s)
Winter field survival FN (t)
Vernalization response (days to anthesis) FN (t)
Groat percent KO (m)
BCD1049
BCD1280A
Days to anthesis KO (b)
Plant height KO (b)
Crown rust reaction, *Pcq2* OM (u)
